# A two-tiered unsupervised clustering approach for drug repositioning through heterogeneous data integration

**DOI:** 10.1186/s12859-018-2123-4

**Published:** 2018-04-11

**Authors:** Pathima Nusrath Hameed, Karin Verspoor, Snezana Kusljic, Saman Halgamuge

**Affiliations:** 1 Department of Mechanical Engineering, University of Melbourne, Parkville, Melbourne, 3010 Australia; 2 Data61, Victoria Research Lab, West Melbourne, 3003 Australia; 3 Department of Computer Science, University of Ruhuna, Matara, 81000 Sri Lanka; 4 Department of Computing and Information Systems, University of Melbourne, Parkville, Melbourne, 3010 Australia; 5 Department of Nursing, University of Melbourne, Parkville, Melbourne, 3010 Australia; 6 The Florey Institute of Neuroscience and Mental Health, University of Melbourne, Parkville, Melbourne, 3010 Australia; 7 Research School of Engineering, College of Engineering & Computer Science, The Australian National University, Canberra, ACT, 2601 Australia

**Keywords:** Drug repurposing, ATC classification, Drug clustering, Data integration, Heterogeneity

## Abstract

**Background:**

Drug repositioning is the process of identifying new uses for existing drugs. Computational drug repositioning methods can reduce the time, costs and risks of drug development by automating the analysis of the relationships in pharmacology networks. Pharmacology networks are large and heterogeneous. Clustering drugs into small groups can simplify large pharmacology networks, these subgroups can also be used as a starting point for repositioning drugs. In this paper, we propose a two-tiered drug-centric unsupervised clustering approach for drug repositioning, integrating heterogeneous drug data profiles: drug-chemical, drug-disease, drug-gene, drug-protein and drug-side effect relationships.

**Results:**

The proposed drug repositioning approach is threefold; (i) clustering drugs based on their homogeneous profiles using the Growing Self Organizing Map (GSOM); (ii) clustering drugs based on drug-drug relation matrices based on the previous step, considering three state-of-the-art graph clustering methods; and (iii) inferring drug repositioning candidates and assigning a confidence value for each identified candidate. In this paper, we compare our two-tiered clustering approach against two existing heterogeneous data integration approaches with reference to the Anatomical Therapeutic Chemical (ATC) classification, using GSOM. Our approach yields Normalized Mutual Information (NMI) and Standardized Mutual Information (SMI) of 0.66 and 36.11, respectively, while the two existing methods yield NMI of 0.60 and 0.64 and SMI of 22.26 and 33.59. Moreover, the two existing approaches failed to produce useful cluster separations when using graph clustering algorithms while our approach is able to identify useful clusters for drug repositioning. Furthermore, we provide clinical evidence for four predicted results (Chlorthalidone, Indomethacin, Metformin and Thioridazine) to support that our proposed approach can be reliably used to infer ATC code and drug repositioning.

**Conclusion:**

The proposed two-tiered unsupervised clustering approach is suitable for drug clustering and enables heterogeneous data integration. It also enables identifying reliable repositioning drug candidates with reference to ATC therapeutic classification. The repositioning drug candidates identified consistently by multiple clustering algorithms and with high confidence have a higher possibility of being effective repositioning candidates.

**Electronic supplementary material:**

The online version of this article (10.1186/s12859-018-2123-4) contains supplementary material, which is available to authorized users.

## Background

Producing new drugs and marketing them with a complete drug profile is a challenging task as it is a long process and requires a large investment of time and money. Drug repositioning or drug repurposing is the process of identifying new therapeutic uses for existing drugs. It can reduce the time, costs and risks of the traditional drug discovery process [1–4]. The main goal of drug repositioning is to increase the therapeutic use of the existing drugs in the clinical and medical domain. It is believed that drugs having similar profiles are more likely to share similar behavior in presence of similar targets (e.g. proteins) [1, 3–7]. There is also evidence that computational drug repositioning can be improved by heterogeneous data analysis [1, 5, 7–9]. In contrast to laborious in-vivo and in-vitro experiments, computational methods for drug repositioning have become popular as effective and efficient approaches for drug repositioning [1, 3–6]. These methods focus on identifying new uses for existing drugs and finding new associations between other contributing entities like proteins, genes, diseases and side effects to approach this problem.

**Fig. 1:**
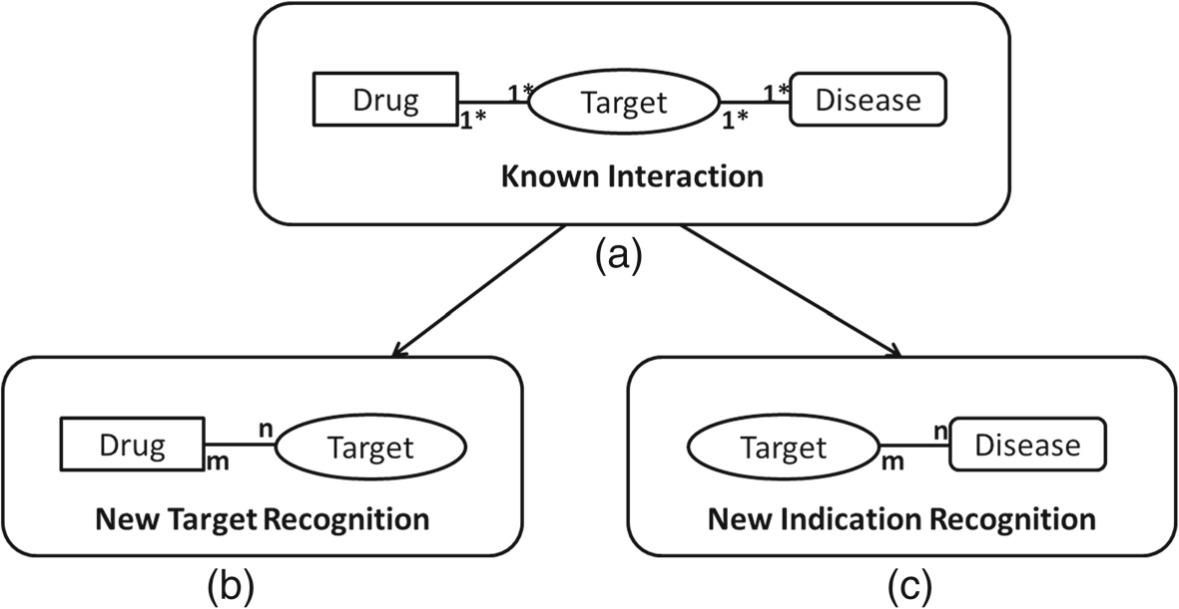
A generalized illustration of two alternative approaches involving in drug repositioning; (**a**), (**b**) and (**c**) represent the known interactions, New Target Recognition and New Indication Recognition, respectively. (The notations 1*-1* and m-n indicate one-or-many and many-to-many relationships, respectively)

There are two main concepts behind drug repositioning: new target recognition and new indication recognition. Figure 1 illustrates a general view of these two drug repositioning concepts. Figure 1a shows the known interactions where each of the drugs is associated with at least one target protein and vice versa; each of the targets is also associated with at least one disease and vice versa. Figures 1b and c show new target recognition and new indication recognition, respectively. In new target recognition, the objective is to identify novel molecular targets for a given drug while in new indication recognition, the objective is to identify new diseases that may be impacted by one of the existing targets of the drug. Computational methods like network based inferencing [1, 5, 6, 8, 10], machine learning [2, 11, 12], and text mining approaches [13, 14] are widely used for drug repositioning. In recent computational approaches, the Anatomical Therapeutic Chemical (ATC) classification system [15] is considered as an intermediate source to identify useful drug repositioning candidates where the ATC therapeutic classes are used to identify repositioning candidates [9, 11, 16]. Every repositioning candidate identified by computational models may not be directly applicable in clinical practice. However, the outcomes of the computational models may enable prioritizing repositioning candidates for in-vivo/in-vitro analysis.

Pharmacological data can be represented in homogeneous or heterogeneous graphs/networks. Therefore, most of the drug repositioning approaches can be seen as hybrid methods of graph/network theory concepts and machine learning [5, 8–10, 12]. Graph clustering is such hybrid approach where graphs of homogeneous and heterogeneous objects can be grouped into small clusters based on their associations. Since pharmacology networks are large and complex, partitioning large networks produces an abstraction which simplifies their complex interaction structure. Realizing the importance of simplifying drug-data network, research [2, 8, 10, 17, 18] has approached partitioning pharmacological networks using various graph theory concepts.

Yildirim et al. [8] focused on combining heterogeneous data using drug-target and disease-gene interactions employing bipartite graph projections while Hartsperger et al. [19] demonstrated the importance of fuzzy clustering for arranging the biological entities like disease, gene and proteins in a meaningful weighted k-partite graph. Moreover, Klamt et al. [20] demonstrates graph transformations such as graph projection methods would lead to information loss. In contrast, Yaminishi et al. [5] investigated a supervised bipartite graph inferencing approach by integrating chemical and pharmacological properties. Campillos et al. [18] suggested a probability theoretic approach to integrate chemical and pharmaceutical properties.

Napolitano et al. [2] proposed useful drug reclassifications for ATC classification using supervised machine learning. They integrated drug-chemical, drug-gene and drug-protein representations and obtained classification accuracy of 78%. But, integrating pharmacological concepts is also important when focusing drug repositioning using ATC classification. In general, taking second/higher order derivatives of objects is a popular method for highlighting special features. Lee et al. [9] proposed that drug groups (DG) having common DG-DG interaction partners would share similar drug mechanisms and they have proposed Molecular Complex Detection (MCODE) algorithm for module detection in DG-DG interaction network. They investigated clustering DG-DG interactions in relation to ATC classification and they believe DG-DG interactions would be useful in describing the mechanisms and the features of drugs.

**The importance of heterogeneous data integration** In preliminary investigations of drug repositioning, computational models for pharmacological data have been developed using homogeneous components such as disease, symptoms, side effects, chemical structures, proteins and genes. But, each homogeneous component has its own pros and cons [1]. Although many findings acknowledge the benefits of phenome space properties like disease and side effects [18, 21], chemical structures are also important to make predictions. Different drug characterizations may lead to identifying various repositioning candidates based on different aspects. Hence, combining the results of different drug characterizations can lead to identifying reliable repositioning candidates. Recent studies have focused on the development of novel, efficient and reliable computational models to improve the final predictions using heterogeneous data integration [1, 2, 5, 8, 9].

In early research, symptom similarities have been employed to analyze disease similarities and in turn to identify new uses for existing drugs [22]. However, it was realized that symptom-based similarities alone are inadequate to predict new therapeutic uses for existing drugs. Consequently, mRNA expression and protein-protein interaction networks have been used in investigating disease similarities [6]. Campillos et al. [18] demonstrated the significance of using side effect similarity for drug repositioning. Even though side effect similarities can be used to link the interactions between drugs and targets, there are certain limitations as well. Some side effects arise due to hormonal changes of the body. Also, side effects may require a long time to observe and construct a strong drug-side effect profile. Hence, it cannot be directly applied to the newly arrived drugs without an explicit drug profile. Since many side effects are common among various drugs, data redundancy is another problem in the side effect domain.

Campillos et al. [18] and Dudley et al. [1] have also investigated the impact of chemical similarities for drug repositioning. They found that using chemical structural similarities alone is insufficient as drugs undergo metabolic transformations and pharmacokinetic transformations. Therefore, studying the mechanism of action of a drug is encouraged. Using connectivity maps to construct the molecular activity profiles based on gene expression has been considered as a better approach as it simplifies drug comparisons. However, a molecular activity similarity based approach may not be very accurate as many disease conditions involve in more than one molecular activity. Moreover, gene expression profiles may be generated under different conditions such as different doses, time durations, different disease stages and ages. Therefore, considering gene expression alone may result in poor performance.

Yamanishi et al. [5] have demonstrated the importance of spanning chemical, genomic and pharmacological space features in discovering new drug-target interactions using supervised bipartite graph inference. They found that pharmacological effect similarities more strongly correlate with new predictions than chemical similarities. Moreover, they proposed a two-step strategy to combine chemical, genomic and pharmacological properties using supervised bipartite graph learning and hence obtained reliable drug-target associations.

In-silico drug repositioning has become very popular during the last decade as it contributes to accelerating drug development and drug discovery. Moreover, recent research has identified heterogeneous data integration as important for obtaining reliable predictions. However, introducing heterogeneous data types increases the complexity of data representation and the number of features. Therefore, network partitioning or clustering methods can be used to simplify large and complex pharmacology data and predictions can be efficiently made on identified subgroups [8–10, 19, 23]. Consensus clustering is a method used for ensemble clustering [24]. It has been introduced to overcome the limitations of basic clustering algorithms. It can also be considered as a method to integrate multiple sources. However, the existing consensus clustering algorithms require the number of clusters to be defined in advance. In this study, we propose a two-tiered clustering approach for drug repositioning inspired by consensus clustering. Here, we selected clustering algorithms which could be employed without any prior knowledge about drug clusters.

Pharmacology networks are large and heterogeneous; drugs can be considered as the main hubs in these networks. The main objective of this study is to construct a consistent computational model for drug repositioning through heterogeneous data integration. Drug-chemical, drug-gene, drug-protein, drug-disease and drug-side effect relationships are useful to represent different aspects of drugs such as chemical, biological and phenome characteristics, respectively. We therefore cluster drugs based on their heterogeneous associations. Specifically, we apply clustering of drugs to simplify the large drug-centric pharmacology networks. In this study, we propose a two-tiered clustering approach, an unsupervised learning approach for drug repositioning via ATC classification. This proposed approach enables clustering drugs based on heterogeneous data integration which is used as the drug similarity model for drug repositioning. Hence, the final clustering is an overall solution that groups similar drugs using a variety of drug characteristics. The identified drug clusters are compared against already published ATC classification to infer useful repositioning candidates. The identified drug clusters can be used as a source to understand drug-drug similarities as well as drug-group similarities.

As illustrated in Fig. 1, new target recognition and new indication recognition are two typical ways of approaching drug repositioning. Even though the use of ATC classification is popular in the input space to determine anatomical/therapeutic/chemical features of drugs [25–27], little research directly focuses on drug repositioning by ATC classification [2, 16, 28]. Recent research [2, 28] limited their studies only for the drugs that already possess an ATC code. Recently, Sun et al. [16] proposed a semi-supervised learning approach based on a physarum-inspired prize-collecting steiner tree approach, for drug repositioning. It applies to infer a single subnetwork at a time, where ATC-C class is used to reposition drugs for Cardiovascular diseases.

This paper fills the gap with a purely unsupervised learning approach by heterogeneous data integration where ATC classification is employed for large-scaled drug repositioning of drugs with and without assigned ATC class. This study also presents a confidence measure which is used to determine the significance of the inferred repositioning candidates. Moreover, the significance of findings arising from this study is twofold; (i) correctly profile and suggest therapeutic indication for drugs that do not possess the ATC code; (ii) flag potential of some drugs to be used for other therapeutic purposes. Furthermore, we provide clinical evidence for four predicted results (Chlorthalidone, Indomethacin, Metformin and Thioridazine) to support that our proposed approach can be reliably used to infer ATC code and drug repositioning.

## Methods

**Fig. 2:**
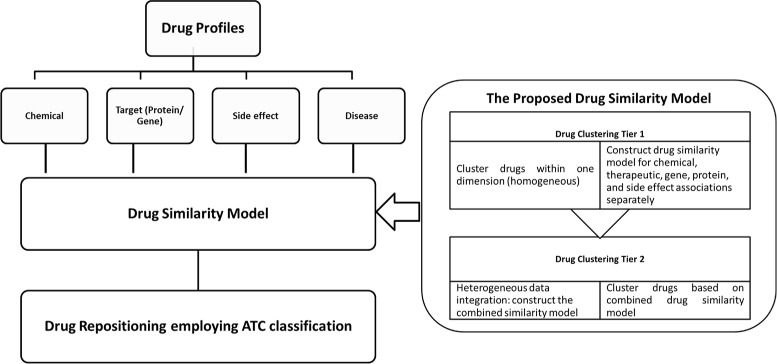
The proposed approach

As explained in “Background” section, drug repositioning candidates can be identified by analyzing drug-drug similarities. This study proposes an unsupervised two-tiered clustering model to identifying drug similarities based on heterogeneous drug characteristics. Figure 2 illustrates the main steps of the proposed approach. A two-tiered clustering approach is proposed to build the drug similarity model for drug repositioning. In Drug Clustering Tier 1, clustering is performed based on drugs’ chemical, therapeutic, gene, protein and side effect associations separately to illustrate how close two drugs are, along each dimension. Drug clustering Tier 2 is a heterogeneous data integration phase, in which the results of Drug Clustering Tier 1 are combined to produce an overall similarity that considers all aspects of the drug similarity. Drug repositioning is carried out employing ATC classification for the drug clusters identified at Drug Clustering Tier 2. The therapeutic classification of the ATC classification is used to label each cluster from which we identify plausible repositioning candidates.

The particular drug profile leading to identifying similar therapeutic uses may vary from drug to drug; choosing an appropriate representation for drug repositioning is challenging. Therefore, making a similarity decision based on heterogeneous drug profiles such as chemical, disease, genes, proteins and side effect is worthwhile. Moreover, some dimensions can be incomplete. If the data in one drug profile is inaccurate or incomplete, it may be compensated by better data in other drug profiles. Therefore, making the final conclusions based on consolidated heterogeneous data enables less errors. ATC classification is used as the gold standard reference classification. We expect that drugs that are in the same ATC class should be clustered together and hence we can use this to validate our clusters.

In “Data” section, the drug data and their ATC classification codes used in this study are explained. In “The proposed approach” section, we explain the selected clustering algorithms, the proposed two-tiered clustering approach, the evaluation process for the identified drug clusters and the computation of confidence measure.

### Data

#### Drug profiles

We use five different homogeneous drug profiles where four of them are obtained from DyDruma [29] database: drug-chemical, drug-therapeutic, drug-protein and drug-side effect profiles. We obtained the KEGG gene data used in Wu et al. [10] to represent drug-gene relationships. This allows us to link drug associations in the genomic space, adding a fifth homogeneous drug dimension. These drug profiles are represented as binary associations where values 1 and 0 represent the presence and absence of a particular feature, respectively.

- **drug-chemical features** [881]: Each drug is associated to relevant chemical fingerprints, based on the 881 fingerprints (2D chemical structures) defined by PubChem [30]. We assume one feature for each fingerprint. If a drug contains a given structural fingerprint, the corresponding feature will have a value of 1.
- **drug-therapeutic features** [719]: The therapeutic uses of the drugs have been obtained by extracting treatment relationships between drugs and diseases from the Unified Medical Language System (UMLS) [31]. These are the treatment relationships between drugs and diseases from the National Drug File-Reference Terminology.
- **drug-protein features** [775]: The target protein information of drugs has been obtained from Drugbank [32] and they have been mapped using UniProt Knowledgebase [33].
- **drug-side effect features** [1385]: The drug-side effect information has been extracted from the SIDER database [34] which uses UMLS library to map the side effect keywords.
- **drug-gene features** [1504]: We constructed a drug-gene binary profile for the 1504 KEGG gene data used in Wu et al. [10] to represent drug-gene relationships.

These five sources have 417 drugs in common. The drug profiles of the selected drugs are available at https://github.com/fathimanush786/two_tiered_clustrering_data.

#### ATC classification

As defined by World Health Organization, the Anatomical Therapeutic Chemical (ATC) classification [15] captures the pharmacodynamic properties of drugs. This resource uses active ingredients of drugs as well as their anatomical, therapeutic and chemical properties when constructing the classification system. ATC is a five level classification system. The first level classification is based on the anatomical group; it contains 14 groups. The second level classification is based on pharmacological/therapeutic subgroups. The third and fourth levels denote chemical/pharmacological/therapeutic subgroups and the fifth level refers to the chemical substance. Some drugs have been categorized into multiple classes. These classifications may also be updated based on new research findings. We obtained ATC classes for 405 drugs out of the 417 selected drugs and 12 drugs had not yet been assigned into ATC classification. We focus on classifying only up to the second (therapeutic) level as our broader goal is to infer new therapeutic uses for existing drugs. We observe 66 unique classes at ATC second level classification for these 405 drugs. These 66 classes are used as the reference clustering to evaluate the performance of the drug clusters identified by our method. The ATC classification of the selected 417 drugs are available at https://github.com/fathimanush786/two_tiered_clustrering_data.

### The proposed approach

Our two-tiered unsupervised clustering model is proposed as a similarity model to identify drugs with closer relationships. Unsupervised clustering is an approach to grouping similar objects together without any prior knowledge of their class labels. Objects that are in a given cluster should demonstrate higher similarity to each other and relatively higher dissimilarity with the objects in other clusters. In general, clustering is popular as a powerful technique which can identify useful patterns in an unsupervised learning environment. There are numerous clustering algorithms that have been proposed. But, there is no acknowledged single preferred algorithm. Each algorithm has its own pros and cons. However, scalability, robustness, handling high dimensional features, speed, intrinsic nature, adaptability and preserving topological order like properties are some interesting characteristics which we have considered in this context.

In the context of drug data, we can apply clustering algorithms by adopting a representation of each drug that allows drug similarity to be computed. We propose a two-tiered clustering approach to cluster drugs into smaller groups based on heterogeneous data integration. We employ four clustering algorithms for partitioning the pharmacology network. We employ Growing Self Organizing Map (GSOM) [35, 36] which is a vector-based clustering algorithm and three state-of-the-art graph clustering algorithms: Markov Clustering (MCL) algorithm [37, 38], Clustering with Overlapping Neighborhood Expansion (ClusterONE) [39] and Molecular Complex Detection (MCODE) [40]. In general, these selected clustering algorithms can be applied without any prior knowledge about the number of classes, which is more useful in this context. We compare the performance of clusters identified by each algorithm to the classes of the ATC classification. We demonstrate the performance evaluation of drug clustering using internal and external evaluation measures. The identified drug clusters are used for drug repositioning via ATC classification.

#### Selected clustering algorithms

**GSOM** Growing Self Organizing Map (GSOM) [35, 36] is an extended version of Self-organization map (SOM) [41] which is a popular vector-based clustering algorithm, capable of handling large-scale and high dimensional features. It is popular for its growing nature while preserving the topological order. It also demonstrates an emergent nature where it starts with one node and it assigns data points considering the shortest Euclidean distance. Spread factor is the parameter which controls the granularity of the cluster map. Smaller spread factor results in a fewer number of nodes in the GSOM map while larger spread factor enables a high growth of the GSOM map.

**ClusterONE** Clustering with Overlapping Neighborhood Expansion (ClusterONE) [39] is a graph partitioning algorithm initially proposed for identifying overlapping protein modules in protein-protein interaction network and also used in a drug repositioning application [10]. It uses a seeded growing concept where it starts with one vertex and it adds or removes vertices in greedy approach to achieve better cluster separations with high cohesiveness.

**MCL** Markov Clustering (MCL) [37, 38] algorithm is another graph clustering algorithm which is also widely used as a protein module detection algorithm for large protein networks. It has been used in a recent drug repositioning application as well [23]. It is popular for its scalability, fast, intrinsic, adaptable and emergent nature. It uses a stochastic flow simulation based concept to partition graphs/networks. It’s parameter ‘inflation’ can be used to control the number of clusters where smaller inflation produces lower granularity with large clusters.

**MCODE** The Molecular Complex Detection (MCODE) [40] algorithm includes three stages: vertex weighting, complex prediction and optionally post-processing to filter or add inputs in the resulting complexes by certain connectivity criteria (haircut and fluffing). MCODE uses a method based on clustering coefficient when assigning weights for vertices. The vertex weight threshold parameter can be used to define the density of the resulting complex. A threshold that is closer to the weight of the seed vertex identifies a smaller, denser network region around the seed vertex.

#### Drug Clustering Tier 1

**Fig. 3:**
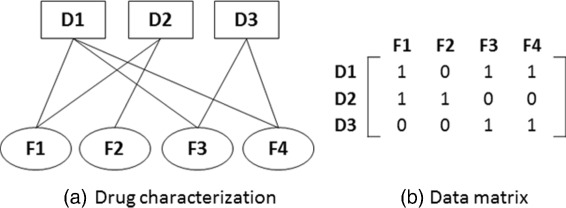
Drug-feature associations could capture in a bipartite graph as shown on (**a**) and its corresponding adjacency matrix is shown on (**b**). D(1,2,3) denotes the drugs while F(1,2,3,4) denotes the features such as chemical, disease, protein and side effect

According to the fundamental graph theory concepts, any drug-feature/drug-drug associations can be represented in two ways; (i) graph representation and (ii) vector/matrix representation. Therefore, we can obtain an adjacency matrix to represent the drug-feature associations as shown in Fig. 3. An adjacency matrix demonstrates which vertices/nodes of a graph/network are adjacent to which other vertices/nodes. In this manner, we have adjacency matrices (data matrices) of 417 ×881, 417 ×719, 417 ×1504, 417 ×775 and 417 ×1385 for each drug-chemical, drug-disease, drug-genes, drug-protein and drug-side effect associations, respectively. Then, we cluster drugs with respect to these independent homogeneous features using GSOM algorithm.

#### Drug Clustering Tier 2

**Fig. 4:**
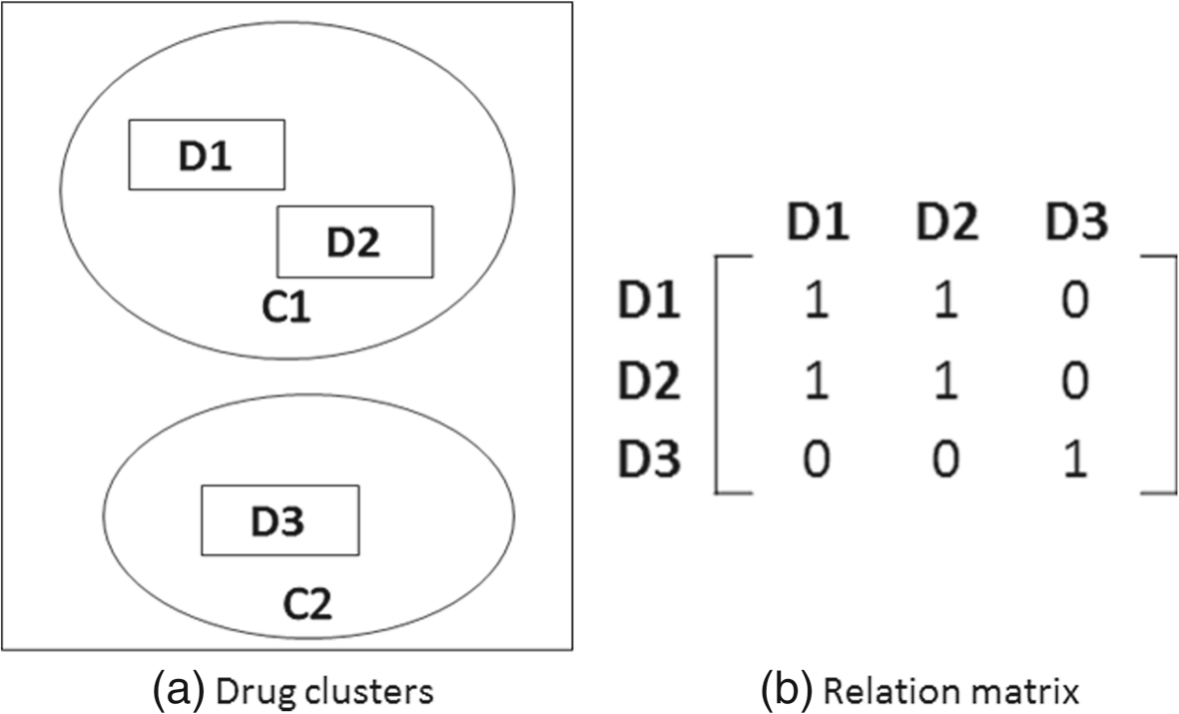
**a** illustrates drug clusters while (**b**) illustrates its corresponding drug-drug associations. D(1,2,3) and C(1,2) denote the drugs and the clusters, respectively

The clustering solutions obtained from Drug Clustering Tier 1 are used to derive drug-drug relation (DDR) matrices. Hence, we produce one DDR matrix per dimension considering their Tier 1 cluster assignments. We then cluster drugs based on combining these individual DDR matrices in order to capture overall drug similarities of aggregated features used in Tier 1. Figure 4 illustrates the mechanism for deriving the DDR matrix using drug clusters (from Drug Clustering Tier 1). We construct five DDR matrices for chemical, disease, gene, protein and side effects separately, based on the individual Tier 1 clustering for each type of feature. We then integrate the DDR matrices of Tier 1 clustering into a single relation matrix by averaging the individual DDR matrices. The averaged relation matrix is used to cluster drugs. By performing this second round of clustering, we aim to improve the reliability of the drug clustering. We employ ClusterONE, MCL, MCODE as well as GSOM in Drug Clustering Tier 2.

### Alternative approaches

#### Concatenating all features into a single vector

A straightforward approach to integrating heterogeneous features is to concatenate all individual features into a single vector [16, 42]. Let *D* be a set of drugs { *D*_1_,*D*_2_,*D*_3_,…,*D*_*n*_} where *C*={ *C*_1_,*C*_2_,*C*_3_,…*C*_*k*_} be the binary vector of chemical features of drug *D*_*i*_ and *T*={ *T*_1_,*T*_2_,*T*_3_,…,*T*_*l*_} be the binary vector of therapeutic features of drug *D*_*i*_. Then, we can construct a heterogeneous data representation (*H*_*y*_) of chemical and therapeutic features by concatenating features from different domains where *H*_*y*_= {*C*_1_,*C*_2_,*C*_3_,...*C*_*k*_,*T*_1_,*T*_2_,*T*_3_,...,*T*_*l*_} be the heterogeneous data integrated binary vector of drug *D*_*i*_, for *i*∈1,2,3,…,*n*. Similarly, we can extend this to integrate drug profiles of multiple domains.

#### Averaging summarized pairwise similarities

Another way of integrating heterogeneous features is to average the similarity measure for each member of a drug pair according to each individual type of feature, to obtain a single summary similarity score [2]. Jaccard coefficient is widely used to obtain the similarity measure between two drugs. Let *Sim*_*C*_(*D*_*i*_,*D*_*j*_) and *Sim*_*T*_(*D*_*i*_,*D*_*j*_) be the chemical and therapeutic similarity measures of a pair of drugs *D*_*i*_ and *D*_*j*_, respectively. Then, we can construct a heterogeneous data representation (*H*_*z*_) by averaging *Sim*_*C*_ and *Sim*_*T*_ where *H*_*z*_ = $\frac {\textit {Sim}_{C} + \textit {Sim}_{T}}{2}$ which would lead to provide a nxn square DDR matrix (where n is the number of drugs). We can extend this to integrate drug profiles in terms of more than two dimensions of similarity.

### Evaluation

#### Internal evaluation

The objective of internal validation is to examine the *compactness/cohesion* and the *separation* of the clusters [43]. There are various internal validation measures and they are variations of these two. But, there is no acknowledged measurement of choice. Silhouette analysis is used as an internal evaluation technique to assess the consistency within a cluster/class because it takes both *compactness/cohesion* and *separation* into account. Moreover, Silhouette can be interpreted using visual aids for in-depth analysis.

Silhouette analysis is used as an internal evaluation technique to assess the consistency within a cluster/class [44, 45]. It measures the similarity of an object to its own cluster/class compared to the other clusters/classes. If the object has a greater similarity to its own cluster/class than to its other clusters/classes, the Silhouette value would be +1 and if the object has greater dissimilarity to its own cluster/class than to the other clusters/classes, the Silhouette value would be -1. The following equation defines the Silhouette measure for an object *i*:

1 $$ Silhouette (i)= \frac{b(i)-a(i)}{max\{a(i),b(i)\}}  $$

where *a(i)* and *b(i)* are the dissimilarity of the object *i* to its own cluster/class and the dissimilarity of the object *i* to the other clusters/classes.

#### External evaluation

We employed ATC classification to compare the performance of our two-tiered clustering approach as well as the performance the clustering algorithms used in this study. We selected adjusted measures: Normalized Mutual Information (NMI) [24] and Standardized Mutual Information (SMI) [46] to evaluate the identified clusters with reference to ATC classification. These are information theoretic measures derived based on mutual information. NMI provides a normalized measure using mutual information where it ranges between 0 and 1. SMI provides a statistical adjustment for the mutual information which is beneficial in adjusting selection bias and to increase the interpretability. SMI further reduces the bias in clustering comparisons towards selecting clusterings with more clusters and where clustering involves fewer data points. The upper bound of SMI varies based on the used reference clustering, however, higher SMI value indicates better clustering. The equations for NMI [24] and SMI [46] to compare clustering solutions U and V are shown below:

2 $$ NMI_{sqrt} (U,V) = \frac{(MI(U,V))}{\sqrt{H(U)H(V)}}  $$

3 $$ SMI (U,V) = \frac{MI(U,V)-E\left[MI(U,V)\right]}{\sqrt{var(MI(U,V))}}  $$

where *MI* is the mutual information, *H* is the associated entropy value, *E* is the expected value and *var* is the variance.

#### Assigning confidence measure

Since a drug can belong to more than one ATC class, identifying drug clusters with 100% pure ATC class is challenging. Therefore, we identify the majority class for each drug cluster and assign a confidence measure for each identified majority class. Then, we predict the identified majority class as a reclassification for the drug/s belongs to minority class/s with the confidence measure as defined by the following equation:

4 $$ {\begin{aligned} confidence_{i}= \frac{number\ of\ drugs\ belong\ to\ the\ major\ ATC\ class\ of\ cluster\ i}{total\ number\ of\ drugs\ of\ cluster\ i} \end{aligned}}  $$

where *i* is the cluster number/id. Hence, we can employ the confidence measure to filter the most useful repositioning candidates.

### Drug repositioning via ATC therapeutic classes

As explained in “ATC classification” section, ATC classification consists of five levels where the second level determines drug’s therapeutic uses/properties. In this study, we approach drug repositioning by identifying plausible new ATC therapeutic (second level) classes for existing drugs. Identifying the drug’s second level classification implies its therapeutic uses. We believe reclassification of drugs into ATC therapeutic (second level) class would enable inferring repositioning candidates.

The use of unsupervised clustering methods enables grouping of drugs without any prior knowledge of ATC classes. We expect that drugs in the same cluster will demonstrate similar characteristics while being relatively dissimilar to drugs in other clusters. Therefore, new drug-drug similarities can be identified by analyzing the drug clusters. The identified new drug-drug similarities lead to propose classification of drugs into new ATC therapeutic (second level) classes. These proposals are inferred based on the majority ATC class associated with each cluster. Classes with higher confidence (see “Assigning confidence measure” section) can be prioritized for reclassification. Since we compare the drug clustering solutions with reference to ATC therapeutic (second level) classes, this reclassification step enables inference of repositioning candidates via ATC therapeutic classes.

## Results

### Drug Clustering Tier 1

First, we clustered drugs based on their individual, homogeneous properties; chemical, disease, gene, protein and side effects. We employed GSOM to cluster drugs in Drug Clustering Tier 1 because it is a vector based clustering algorithm. In this study, we used the GSOM implementation of Chan et al. [47] because of its convenient visual aids for cluster analysis. As mentioned in “Selected clustering algorithms” section, we tuned the parameter, spread factor (SF), to obtain GSOM maps of different sizes. As a result, we obtained GSOM maps of 68 (SF =0.0001), 69 (SF =0.25), 66 (SF =0.8), 63 (SF =0.2) and 63 (SF =0.001) nodes for chemical, disease, gene, protein and side effects profiles, respectively. Out of 417 drugs, 405 drugs have already classified into at least one ATC class. Moreover, we noticed 66 unique ATC classes (2 ^*nd*^ level ATC classification) relating these 405 drugs. We evaluated drug clustering solutions for these 405 drugs with reference to the ATC classification.

Table 1 shows NMI and SMI values for Drug Clustering Tier 1. Accordingly, the NMI varies between 0.46 and 0.68 and SMI varies between 2.91 and 39.33. As of ATC classification, anatomical and therapeutic features are considered in its first two classification levels. Hence, drug clustering using disease and protein profiles demonstrate relatively higher NMI and SMI. The NMI and SMI of chemical and side effect profiles are relatively lower than disease and protein profiles as they are considered in the third, fourth and fifth levels of ATC classification. On the other hand, clustering solution on gene profiles shows the least closeness to ATC classification as this type of information is not considered in ATC classification system. Unlike NMI where the upper bound is always 1.0, the upper bound for SMI depends on the choice of reference clustering; the upper bound for ATC reference clustering is 98.18. Notably, the ranking order of these clustering solutions is consistent for both NMI and SMI.

**Table 1:** Performance assessment of Drug clustering Tier 1

Drug profiles	NMI	SMI
Chemical	0.59	20.09
Disease	0.68	39.33
Gene	0.46	2.91
Protein	0.63	30.38
Side Effect	0.58	21.07

Approximately 16% of the drugs (out of 405 drugs) are assigned to multiple classes. Therefore, we randomly selected one ATC class for those drugs having multiple classes when constructing the reference class list. Additional file 1: Figure S1 corresponds to the Silhouette analysis for chemical, disease, gene, protein and side effect profiles, respectively. It is clear that most of the drugs show negative Silhouette values, illustrating higher variations within ATC classes. The mean Silhouette value of ATC classification based on chemical, disease, gene, protein and side effects are − 0.31, − 0.06, − 0.49, − 0.25 and − 0.33, respectively. However, disease profiles provide relatively greater consistency with the ATC classification compared to other drug profiles.

Moreover, the Silhouette analysis on GSOM identified drug clusters demonstrates relatively higher Silhouette values than ATC classification where the mean Silhouette value for chemical, disease, gene, protein and side effect using GSOM algorithm are 0.13, 0.09, 0.22, 0.15 and -0.07, respectively which are relatively higher than ATC classification (see Additional file 1: Figure S2 for the Silhouette analysis).

Furthermore, we examined the closeness of the clustering solutions between different drug properties used in this study. In Tables 2 and 3, we show the clustering comparison between different drug profiles using NMI and SMI, respectively. In these tables, we compare the drug clusters generated by one type of drug profile with the drug clusters generated by another type of drug profile. For instance, drug clusters generated using chemical properties are compared against drug clusters generated by disease, gene, protein and side effect profiles. According to Table 2, NMI of 0.55, 0.48, 0.59 and 0.56 have been observed between drug clusters generated by chemical profile and drug clusters generated by disease, gene, protein and side effect, respectively. Similarly, according to Table 3, SMI of 12.71, 0.50, 20.85 and 9.98 have been observed between drug clusters generated by chemical profile and drug clusters generated by disease, gene, protein and side effect, respectively.

**Table 2:** Drug clustering comparison between drug profiles based on Normalized Mutual Information (NMI)

	Disease	Gene	Protein	Side effect
Chemical	0.55	0.48	0.59	0.56
Disease		0.45	0.59	0.55
Gene			0.45	0.47
Side effect				0.56

**Table 3:** Drug clustering comparison between drug profiles based on Standardized Mutual Information (SMI)

	Disease	Gene	Protein	Side effect
Chemical	12.71	0.50	20.85	9.98
Disease		1.08	22.06	14.89
Gene			2.58	0.89
Side effect				16.43

According to NMI, drug clusters of chemical profiles, disease profiles, protein profiles and side effect profiles show relatively closer similarities where they vary between 0.55 and 0.59. On the other hand, the highest SMI is noticed between clusters of disease and protein profiles. Notably, drug clusters of gene profiles are relatively far away from other drug clustering solutions. This deviation might have caused due the highly sparse nature of the gene profiles. Moreover, the clusters identified by gene profiles lie relatively very far away from ATC classification than the other clusters. Therefore, we selected chemical, disease, protein and side effect profiles for further analysis to identify drug repositioning candidates using ATC classification.

We identified a set of 26 pairs of drugs (see Additional file 2) which occur together in each drug cluster, generated based on individual chemical, disease, protein and side effect profiles. 25 out of these 26 drug pairs are assigned to the same ATC class (second level), indicating meaningfulness of the identified drug clusters. Fluphenazine and Thioridazine are also identified in the same cluster in all four clustering solutions. However, Thioridazine does not belong to any of the ATC classes while Fluphenazine belongs to ATC class N05 (-psycholeptics). Therefore, we believe Thioridazine may share similar drug profile as of Fluphenazine and we propose to classify Thioridazine into N05 (-psycholeptics).

### Drug Clustering Tier 2

As explained above, we employed the four drug clusterings generated based on chemical, disease, protein and side effect profiles in Drug Clustering Tier 2. We constructed four DDR matrices based on these four identified drug clustering solutions (as explained in “Drug Clustering Tier 2” section in “Methods” section). We propose merging of these DDR matrices into a single matrix as a way of heterogeneous data integration. The merged DDR matrix can be constructed by giving equal importance to each of the drug clusterings or by ranking the drug clusterings based on different evaluation measures such as NMI and SMI. However, there is no single type of homogeneous drug characteristics identified to provide an efficient and effective drug classification or drug repositioning [1]. Giving equal importance to each of the drug clusterings, we constructed a heterogeneous DDR matrix by averaging the four DDR matrices.

We used the averaged DDR matrix to identify drug clusters, employing the graph clustering algorithms: ClusterONE, MCODE and MCL as well as the GSOM algorithm. In this study, we used ClusterONE, MCL and MCODE implementations available in MATLAB Systems Biology and Evolution Toolbox (SBEToolbox) [48]. We obtained a GSOM map of 63 nodes when SF is 0.2. We identified 64 clusters using MCODE when the threshold parameter is set to 0.9. Increasing the threshold from (0, 0.9] increased the number of clusters. We identified 66 clusters using MCL when inflation parameter is set to 0.048. The number of clusters increases when the inflation parameter is increased. We obtained two clustering solutions; CL1 _*I*_ and CL1 _*II*_ employing ClusterONE. CL1 _*I*_ is obtained when the density parameter is set to 0.6 and ‘nodes’ is used as the seed method while CL1 _*II*_ is obtained when the density parameter is set to 0.8 and ‘unused-nodes’ is used as the seed method. CL1 _*I*_ resulted in 61 clusters including all 417 drugs while CL1 _*II*_ resulted in 58 clusters including only 405 drugs. In ClusterONE, choosing ‘nodes’ as the seed method enables every node to be used as a seed and subgroups smaller than a given density are thrown away.

Table 4 summarizes NMI and SMI values for Drug Clustering Tier 2 using GSOM, MCL, CL1 _*I*_ and MCODE. The GSOM results are relatively higher, measuring NMI and SMI with reference to the ATC classification. The NMI and SMI values of *Drug Clustering Tier 2* are 0.66 and 36.11 while they are 0.68 and 39.33 for disease profiles in *Drug Clustering Tier 1*. However, NMI and SMI values of *Drug Clustering Tier 2* are relatively higher than other four drug profiles. Since we employed ATC therapeutic class as the reference cluster, the results in *Drug Clustering Tier 1* are more favorable towards disease profiles.

**Table 4:** Performance assessment of Drug Clustering Tier 2 using four different clustering algorithms

Algorithm	NMI	SMI
GSOM	0.66	36.11
MCL	0.59	26.49
ClusterONE (*CL*1_*I*_)	0.56	21.37
MCODE	0.52	11.57

We predicted new ATC therapeutic classes based on the identified majority ATC classes in the corresponding clusters which led to reclassification of the existing drugs. In order to filter the most reliable repositioning candidates, we assigned a confidence measure for each prediction (see “Assigning confidence measure” section). We therefore filter the repositioning candidates with high confidence as reliable drug repositioning candidates. The highest confidence measures of the identified major classes are 0.85, 0.83, 0.75 and 0.5 for MCL, ClusterONE, MCODE and GSOM, respectively.

### Comparing the proposed approach against existing methods

We compared the performance of the proposed two-tiered clustering approach against two recently used heterogeneous data integration methods for drug repositioning (see “Alternative approaches” section). Table 5 shows the performance assessments of these three different methods for heterogeneous data integration using GSOM algorithm only. In Drug Clustering Tier 2, GSOM demonstrates NMI and SMI of 0.66 and 36.11, respectively. The all concatenated heterogeneous feature representation method (*H*_*y*_) demonstrates NMI and SMI of 0.60 and 22.26, respectively while averaging summarized heterogeneous (pairwise) similarities (*H*_*z*_) demonstrates NMI and SMI of 0.64 and 33.59, respectively. There is a significant improvement in the proposed approach compared to the alternative method *H*_*y*_. Even though there is no significant improvement in the proposed approach compared to the alternative method *H*_*z*_, *H*_*z*_ fails to produce useful clusters when graph clustering algorithms are used.

**Table 5:** Comparison of the proposed approach against two existing methods for heterogeneous data integration

Method	NMI	SMI
The proposed two-tiered clustering	0.66	36.11
Concatenating all heterogeneous features into a single vector (*H*_*y*_)	0.60	22.26
Averaging summarized heterogeneous (pairwise) similarities (*H*_*z*_)	0.64	33.59

It should be noted that these three heterogeneous data integration methods did not outperform drug clusters identified by disease characteristics where NMI and SMI are 0.68 and 39.33, respectively. Since we employed ATC therapeutic class as the reference cluster, the results in *Drug Clustering Tier 1* are more favorable towards disease profiles. Our proposed approach and alternative method *H*_*z*_ outperformed other three clusterings identified by chemical, protein and side effects profiles in *Drug Clustering Tier 1* whereas alternative method *H*_*y*_ outperformed clusterings identified by chemical and side effects profiles in *Drug Clustering Tier 1*.

The alternative method *H*_*z*_, explained in this study produces a complete graph while the proposed two-tiered clustering approach involves the removal of noisy edges, resulting in a sparse graph for efficient graph clustering. The graph clustering algorithms used in this study are not able to identify useful clusters on the given complete graph where they resulted in producing only one module at all time with all three graph clustering algorithms. Therefore, the proposed two-tiered drug clustering approach as a heterogeneous data integration approach demonstrates better performance and can be considered as a reliable method for both vector-based and graph clustering.

### Drug Repositioning via ATC therapeutic class

We analyzed the drug clusters identified by MCL, MCODE, CL1 _*I*_ and GSOM to infer useful drug repositioning candidates. In Table 6, we show 39 repositioning candidates having a minimum confidence measure of 0.5. Out of these, 4 drugs (Chlorthalidone, Thioridazine, Orphenadrine and Indomethacin) have not been assigned to ATC classification yet. We infer these unclassified Chlorthalidone, Thioridazine, Orphenadrine and Indomethacin for ATC classes C03-diuretics (confidence: 0.83), N05-psycholeptics (confidence: 0.80), R06 -antihistamines (confidence: 0.64) and M01-antiinflammatory and antirheumatic (confidence: 0.57), respectively. Interestingly, in Drug Clustering Tier 1, Thioridazine is inferred to have a similar drug profile as of Fluphenazine which also belongs to ATC class N05-psycholeptics. Moreover, in the predicted repositioning list, Amlodipine is inferred to be repositioned for diseases related to renin-angiotensin system (C09) with the highest confidence measure of 0.85. Even though Amlodipine is not directly classified into C09, fixed combinations of aliskiren,valsartan, hydrochlorothiazide, ACE inhibitors, etc. are already classified in C09 [15].

**Table 6:** The inferred repositioning candidates with higher confidence

Drug name	Cluster ID	Old ATC name	New ATC name	Confidence	Algorithm
Amlodipine	403	C08	C09	0.85	MCL
Chlorthalidone	2		C03	0.83	CL1
Amantadine	51	N04	N05	0.80	CL1
Thioridazine	51		N05	0.80	CL1
Hydroxyzine	30	N05	C09	0.75	MCODE
Cyproheptadine	46	R06	N06	0.70	CL1
Amlodipine	11	C08	C09	0.70	CL1
Carvedilol	11	C07	C09	0.70	CL1
Cetirizine	11	R06	C09	0.70	CL1
Acitretin	414	D05	D10	0.67	MCL
Brinzolamide	48	S01	L02	0.67	MCODE
Orphenadrine	392		R06	0.64	MCL
Clonidine	56	C02, N02, S01	N05	0.62	CL1
Thioridazine	56		N05	0.62	CL1
Dofetilide	399	C01	L02	0.60	MCL
Cyproheptadine	35	R06	N06	0.59	CL1
Guanfacine	35	C02	N06	0.59	CL1
Dipivefrin	44	S01	N05	0.57	CL1
Indomethacin	7		M01	0.57	MCODE
Nicardipine	57	C08	N06	0.57	CL1
Cyproheptadine	4	R06	N06	0.54	CL1
Methadone	4	N07	N06	0.54	CL1
Arsenic Trioxide	4	L01	P01	0.50	MCODE
Atropine	48	A03, S01	N04	0.50	CL1
Atropine	393	A03, S01	N04	0.50	MCL
Dacarbazine	79	L01	A10	0.50	GSOM
Hexachlorophene	350	D08	D05	0.50	MCL
Isocarboxazid	50	N06	N05	0.50	MCODE
Levetiracetam	346	N03	L01	0.50	MCL
Lithium	6	N05	N06	0.50	GSOM
Mercaptopurine	4	L01	P01	0.50	MCODE
Metformin	79	A10	L01	0.50	GSOM
Moexipril	26	C09	C07	0.50	CL1
Mycophenolic Acid	342	L04	N03	0.50	MCL
Phenytoin	66	N03	C01	0.50	GSOM
Tazarotene	350	D05	D08	0.50	MCL
Tolterodine	59	G04	C01	0.50	GSOM
Topotecan	346	L01	N03	0.50	MCL
Zonisamide	342	N03	L04	0.50	MCL

Note: ATC code names are given in Additional file 5

Different algorithms may produce different clustering solutions. However, different algorithms may have similarities too. We identified 79 reclassification predictions which are generated consistently by at least two clustering algorithms or in at least two different clusters (in ClusterONE). Table 7 summarizes 11 reclassification candidates identified consistently by at least two clustering algorithms with relatively high confidence measures (see Additional file 3 for the complete list). ClusterONE algorithm produces overlapping clusters. Therefore, some drugs are assigned to more than one cluster. Table 7 illustrates three drug reclassification candidates (Cyproheptadine, Droperidol and Dolasetron) that are identified by more than one cluster in ClusterONE results.

**Table 7:** Repositioning candidates identified consistently by more than one clustering algorithm

Drug name	Cluster ID	Old ATC name	New ATC name	Confidence	Algorithm
Amlodipine	403	C08	C09	0.85	MCL
Amlodipine	11	C08	C09	0.70	CL1
Cyproheptadine	46	R06	N06	0.70	CL1
Cyproheptadine	35	R06	N06	0.59	CL1
Cyproheptadine	4	R06	N06	0.54	CL1
Cyproheptadine	56	R06	N06	0.25	MCODE
Brinzolamide	48	S01	L02	0.67	MCODE
Brinzolamide	9	S01	L02	0.17	CL1
Atropine	48	A03, S01	N04	0.50	CL1
Atropine	393	A03, S01	N04	0.50	MCL
Atropine	20	A03, S01	N04	0.46	GSOM
Metformin	79	A10	L01	0.50	GSOM
Metformin	21	A10	L01	0.33	MCODE
Mycophenolic Acid	342	L04	N03	0.50	MCL
Mycophenolic Acid	22	L04	N03	0.27	GSOM
Carbamazepine	46	N03	N05	0.43	GSOM
Carbamazepine	42	N03	N05	0.23	CL1
Carbamazepine	20	N03	N05	0.20	MCODE
Carbamazepine	46	N03	N06	0.43	GSOM
Carbamazepine	25	N03	N06	0.27	CL1
Carbamazepine	20	N03	N06	0.20	MCODE
Droperidol	28	N05	N01	0.42	GSOM
Droperidol	359	N05	N03	0.40	MCL
Droperidol	40	N05	N01	0.32	CL1
Droperidol	30	N05	N03	0.17	CL1
Fulvestrant	23	L02	A10	0.42	GSOM
Fulvestrant	13	L02	A10	0.42	CL1
Dolasetron	2	A04	A02	0.40	MCODE
Dolasetron	40	A04	A02	0.20	GSOM
Dolasetron	59	A04	L01	0.20	CL1
Dolasetron	24	A04	L01	0.12	CL1

Note: ATC code names are given in Additional file 5

In this study, ATC classification is considered as the gold standard classification, therefore, we obtained clustering performance with reference to the ATC classification. We used only up to its second level classification as it captures the therapeutic uses. The drugs used in this study include 12 drugs that are not yet assigned into ATC classification. However, our method enables inferring suitable ATC classification for them (see Additional file 4 for the complete list of predictions). Moreover, the inferred new ATC codes of other drugs can be used for drug repositioning. “Clinical significance of our findings” section summarizes some clinical evidence to support these findings. We therefore suggest that cluster-based classification and reclassification into the ATC classification system is a viable method for drug repositioning.

Clustering enables partitioning the large pharmacology network into smaller subgroups and hence simplifies the drug repositioning process. Since drugs can be considered as the main component of the pharmacological networks, drug clustering provides an indirect way of clustering the networks, where associations to related entities (e.g., chemical, target and phenomic) can be incorporated as a basis for clustering. Hence, the proposed two-tiered drug-centric drug clustering can be extended by employing all the other related heterogeneous data at each of the cluster levels. It enables other participating entities to present in more than one cluster. Then, new associations between chemical, target and phenome can be predicted for each of the clusters as well. Moreover, it enables investigation of multiple links connecting drugs and may prove useful for pathway analysis.

### Clinical significance of our findings

The significance of findings arising from this study is twofold; (i) correctly profile and suggest therapeutic indication for drugs that do not possess the ATC code; (ii) flag potential of some drugs to be used for other therapeutic purposes. More interestingly, the inferred therapeutic uses are significantly different to the one for which these drugs were initially developed and trialed. This section summarizes clinical evidence for four findings of this study: Chlorthalidone, Indomethacin, Metformin and Thioridazine.

Our study interestingly inferred the ATC code, C03 and therapeutic use, diuretics, for a drug known as Chlorthalidone (see Table 6), which until now does not belong to the ATC classification. Chlorthalidone is a potent diuretic; a drug that promotes water loss and is currently used in the management of hypertension or high blood pressure and fluid retention associated with heart failure [49]. In fact, Chlorthalidone has better clinical outcome in terms of lowering blood pressure than other more commonly prescribed diuretics [50, 51].

Furthermore, Indomethacin is another drug that does not have an ATC code yet. According to our findings, Indomethacin was indicated to be used as an anti-inflammatory and anti-rheumatic agent (see Table 6). This perfectly matches the clinical situations for which this drug is used; Indomethacin is indicated for managing pain associated with inflammation, rheumatoid arthritis as well as osteoarthritis [52, 53].

Another interesting finding arising from our work relates to Metformin (see Tables 6 and 7). Metformin is used to manage type 2 diabetes and its initial classification was an oral hypoglycaemic, drug that lowers blood sugar level [54]. In the past ten years, Metformin was also found to be therapeutically effective in other diseases such as polycystic ovarian syndrome and metabolic syndrome [55, 56]. Emerging evidence is strongly suggesting that Metformin can now be used as an adjuvant treatment in bowel and prostate cancer due to its antineoplastic properties; can inhibit cancer growth [57, 58]. This is a significant deviation from its original therapeutic use and was correctly inferred in our study by the ATC code L01 and therapeutic class antineoplastic agent.

Furthermore, it is important to mention that our proposed drug repositioning method accurately flagged Thioridazine, a drug that does not possess an existing ATC code, as being psycholeptic agent (see Table 6). Thioridazine is clinically effective in treating patients with schizophrenia since its discovery [59, 60], however, it was withdrawn from the market in 2005 due to its ability to cause toxicity to the heart [61].

## Discussion

### Clustering

Clustering enables partitioning the large pharmacology network into smaller subgroups and hence simplifies the drug repositioning process. Since drugs can be considered as the main component of the pharmacological networks, drug clustering provides an indirect way of clustering the networks, where associations to related entities (e.g., chemical, target and phenomic) can be incorporated as a basis for clustering. Hence, the proposed two-tiered drug-centric drug clustering can be extended by employing all the other related heterogeneous data at each of the cluster levels. It enables other participating entities to present in more than one cluster. Then, new associations between chemical, target and phenome can be predicted for each of the clusters as well. Moreover, it enables investigation of multiple links connecting drugs and may prove useful for pathway analysis.

Clustering algorithms such as k-means, SOM, GSOM and mixture models can be employed in *Drug Clustering Tier 1*. But, K-means, SOM and mixture models are not suitable for drug clustering because the number of clusters and the cluster shapes need to be known and specified in advance [62]. In drug clustering, we cannot expect to have a priori knowledge about the grouping and the cluster shapes. Moreover, higher dimensional feature space in pharmacology data could potentially hinder the efficiency and effectiveness of the machine learning algorithms.

GSOM is well-suited for *Drug Clustering Tier 1 and 2* because it is cable of handling higher dimensional features and the number of clusters is defined automatically. In GSOM, the parameter *spread factor* is used to control the size of the GSOM map or the number of clusters. This *spread factor* does not depend on the dimensionality of the data. Moreover, it preserves the topological order.

MCL, MCODE and ClusterONE algorithms used in *Drug Clustering Tier 2*, are graph clustering algorithms that are popular in the context of pharmacology data analysis. They are also capable of handling high dimensional features and the number of clusters is defined automatically. Unlike vector-based algorithms, these graph clustering algorithms are not appropriate for *Drug Clustering Tier 1* because they result in clustering drugs as well as their corresponding features.

Interestingly, we observed relatively close number of drug clusters in *Drug Clustering Tier 1* and *Drug Clustering Tier 2* after tuning cluster parameters. Table summarizes the parameters and their effect on generating the clusters. The number of clusters generated by GSOM using chemical, disease, gene, protein and side effect profiles are 68, 69, 66, 63 and 63, respectively. In GSOM, parameter *spread factor* can be used to tune the number of clusters. In *Drug Clustering Tier 2*, GSOM, MCL, MCODE and ClusterONE, generated 63, 64, 66 and 61 clusters, respectively. In MCODE, increasing the *threshold* from (0, 0.9] increased the number of clusters. However, further incrementing the *threshold* parameter after 0.9 resulted in a decrement of the number of clusters. Interestingly, we identified 64 clusters using MCODE when the *threshold* parameter is set to 0.9, strengthening our confidence that the number of clusters lies around 64.

Overlapping clustering algorithms may be more suitable for drug clustering as some drugs are used to treat multiple diseases. Moreover, overlapping clusters may enable identifying more repositioning candidates. ClusterONE, MCL and MCODE algorithms used in this study can handle overlapping algorithms. But, in the current analysis, we observed overlapping clusters only from ClusterONE. It should be noted that some repositioning candidates identified by ClusterONE are identified by GSOM, MCL and MCODE as well. Therefore, we believe the repositioning candidates identified by non-overlapping clusters could still be prospective candidates for further in-depth analysis. The repositioning candidates identified by multiple clustering algorithms increase our confidence that they might be interesting.

Since the four clustering algorithms used in this study are capable of handling higher dimensional feature representations, we did not employ dimensionality reduction. Dimensionality reduction techniques may be useful to remove noisy information. But, it is not appropriate for MCL, MCODE and ClusterONE, graph clustering algorithms as they use drug-drug/drug-feature relationships to be the input.

GSOM typically uses Euclidean distance to compute the pairwise distance between input vector and weight vector. The performance of GSOM may be further improved by employing Jaccard similarity or squared Euclidean distance or taking the average distance based on multiple metrics when binary data are used.

### Heterogeneous data integration

Drugs can be explained using various characteristics such as chemical, target and phenomic, etc. The primary objective of heterogeneous/multi-view data integration is to more deeply understand the predictive model and to obtain a consensus solution [63].

Multi-view data integration can be performed at the input/intermediate/output phase [63]. The proposed methods can be seen as a type of multi-view data integration. In the alternative methods *H*_*y*_ and *H*_*z*_, data integration is performed at the input phase and the intermediate phase, respectively, while our presented two-tiered clustering approach performs data integration at the output phase. In our method, the outputs from various individual views are combined and the consensus clustering results are obtained at the second tier. Moreover, in multi-view data integration, a kernel matrix is typically used as an input for kernel classification, regression and clustering [63]. In our study, the clustering results of *Drug Clustering Tier 1* are used to construct the Drug-Drug Relation matrix which can be viewed as a type of kernel matrix. Hence, the proposed method is compatible with existing kernel learning approaches.

Methods such as kernelized Bayesian matrix factorization, random walk methods can be effectively applied on bi-partite graphs as a mean of data integration. Multi-modal deep learning can also be applied to heterogeneous drug data integration where output of each view can be integrated into higher layers [63]. Deep Boltzmann machine would be a suitable approach for drug data clustering where binary data are considered.

### Cluster evaluation

Since drugs can belong to more than one class, the classes induced from the ATC classification can have distantly related drugs which will result in a higher number of false positives in the compared clustering solution. Some drugs in other classes may share higher similarity though they have distinct uses which will also result in a higher number of false positives. Moreover, many ATC classes have very high intra-cluster variations which will result in a higher number of false negatives. Therefore, we cannot expect the identified drug clusters to be highly correlated with ATC classification.

Using Silhouette values to fine tune the parameters would be another approach that we could use when determining the number of clusters. But, it should be noted that the mean Silhouette value of ATC classification based on chemical, disease, gene, protein and side effects are − 0.31, − 0.06, − 0.49, − 0.25 and − 0.33, respectively which illustrates the higher variations within ATC classes. Hence, higher variations within drug clusters are expected. Hence fine tuning the parameters of the clustering algorithms comparison to ATC classification may not be very accurate.

According to Silhouette values of ATC classification, obtaining a clustering close to the ATC classification is challenging due to the large variation within ATC classes, misclassifications and missing information in the ATC classification. The mismatches between the clustering solution and the ATC classification arise due to the identified new drug classes (drug-drug similarities) for the existing drugs. As explained in “Drug repositioning via ATC therapeutic classes” section, the reclassification into ATC therapeutic classes can be interpreted as repositioning opportunities. Also, the clustering solutions enable identifying more useful drug-drug relationships.

External clustering evaluation is an important task though it is challenging. Consequently, various external clustering comparison measures have been proposed. Pair-counting based measures include RI and ARI while MI, NMI and AMI are information theoretic based measures useful to compare clustering solutions against a reference clustering. There is no clear evidence that one measure is superior to another. NMI, AMI and SMI are the adjusted measures for MI and have important benefits [46]. Moreover, SMI is proportional to AMI. We therefore performed clustering evaluation using NMI and SMI.

It is important that the drugs within a cluster are more similar to each other than the other drugs. Our primary objective of this study is not to present a model to predict the ATC classification. Fine tuning parameters against an external reference may not be a better option since our broader focus is to determine the repositioning candidates where they deviate from the current ATC class. Moreover, the false positives predicted by the clusters is not necessarily an undesired result and optimizing clusters for NMI and SMI measure might prevent us from detecting interesting novel clusters or repositioning candidates.

*Drug Clustering Tier 2* achieved 11.9, 4.8, and 13.8% gain in NMI compared to chemical, protein, and side effect, respectively of *Drug Clustering Tier 1* whereas there is a 2.9% loss in NMI compared to disease profile of *Drug Clustering Tier 1*. Since we employed ATC therapeutic class as the reference cluster, the results in *Drug Clustering Tier 1* are more favorable towards disease profiles.

The predicted clusters that do not provide a higher Silhouette value is not necessarily an undesired result. Moreover, not all identified clusters may be useful for drug repositioning. As explained in “Assigning confidence measure” section, we defined a confidence measure so that we can identify the highly probable repositioning candidates. The drug repositioning candidates that are commonly identified by multiple clustering algorithms also have higher probability to be chosen as repositioning candidates. As explained in “Drug Clustering Tier 2” section in “Methods” section, drug-drug relation matrix represents a kernel matrix or similarity matrix, illustrating the similarity means of cluster relationships of drugs. Hence, the drug-drug relational matrix can be straightforwardly incorporated in kernel-based supervised and unsupervised learning methods such as support vector machines, spectral clustering, multiple kernel learning, etc [63, 64].

## Conclusions

Computational drug repositioning provides new strategies for drug development. It has been argued that using heterogeneous features results in better drug repositioning predictions. In this study, we proposed an unsupervised learning approach to achieve drug repositioning by, first, performing drug-centric drug clustering and, second, associating inferred clusters to ATC therapeutic classes based on known drug classifications. Moreover, the proposed two-tiered clustering approach enables drug clustering through heterogeneous data integration. The drug clustering based on core drug features produces clusters that align well with the existing ATC classification levels. The repositioning candidates identified consistently by multiple clustering algorithms and with high confidence have a higher possibility for reliable drug repositioning. Furthermore, the identified drug clusters can be used as an intermediate source to explore drug similarities. The clinical significance of the predicted results also suggests that the proposed two-tiered clustering approach can be safely used to infer new ATC code as well as new therapeutic uses based on the given drug characteristics.

## Additional files

Additional file 1 Silhouette analysis for ATC classification and GSOM clustering. This file includes the figures illustrating the Silhouette values of drugs based on ATC classification and GSOM clustering using chemical, disease, gene, protein and side effect profiles. (PDF 224 kb)

Additional file 2 The 26 pairs of drugs which occur together in Drug Clustering Tier 1. These drug pairs occur together in each drug cluster, generated based on individual chemical, disease, protein and side effect profiles. (PDF 59 kb)

Additional file 3 The repositioning candidates identified consistently by at least two clustering algorithms. This includes the complete list of consistent repositioning candidates, algorithm names and their confidence measures. (PDF 104 kb)

Additional file 4 The complete prediction list. This includes the complete list of predicted new classifications into ATC therapeutic class and their confidence measures. (PDF 892 kb)

Additional file 5 The ATC code list. This includes the ATC codes (Second Level) and the corresponding ATC therapeutic class names. (PDF 363 kb)

## Abbreviations

ATC: Anatomical therapeutic chemical
ClusterONE: Clustering with overlapping neighborhood expansion
DDR: Drug-drug relation
GSOM: Growing self organizing map
MCODE: Molecular complex detection
NMI: Normalized mutual information
SF: Spread factor
SMI: Standardized mutual information
