# Supplementary material for: A two-tiered unsupervised clustering approach for drug repositioning through heterogeneous data integration
Source: BMC Bioinformatics. 2018 Apr 11;19:129. doi: 10.1186/s12859-018-2123-4 (PMC5896044; doi:10.1186/s12859-018-2123-4)
Supplement: Supplementary file 1 — Silhouette analysis for ATC classification and GSOM clustering. This file includes the figures illustrating the Silhouette values of drugs based on ATC classification and GSOM clustering using chemical, disease, gene, protein and side effect profiles. (PDF 224 kb) [file 12859_2018_2123_MOESM1_ESM.pdf]

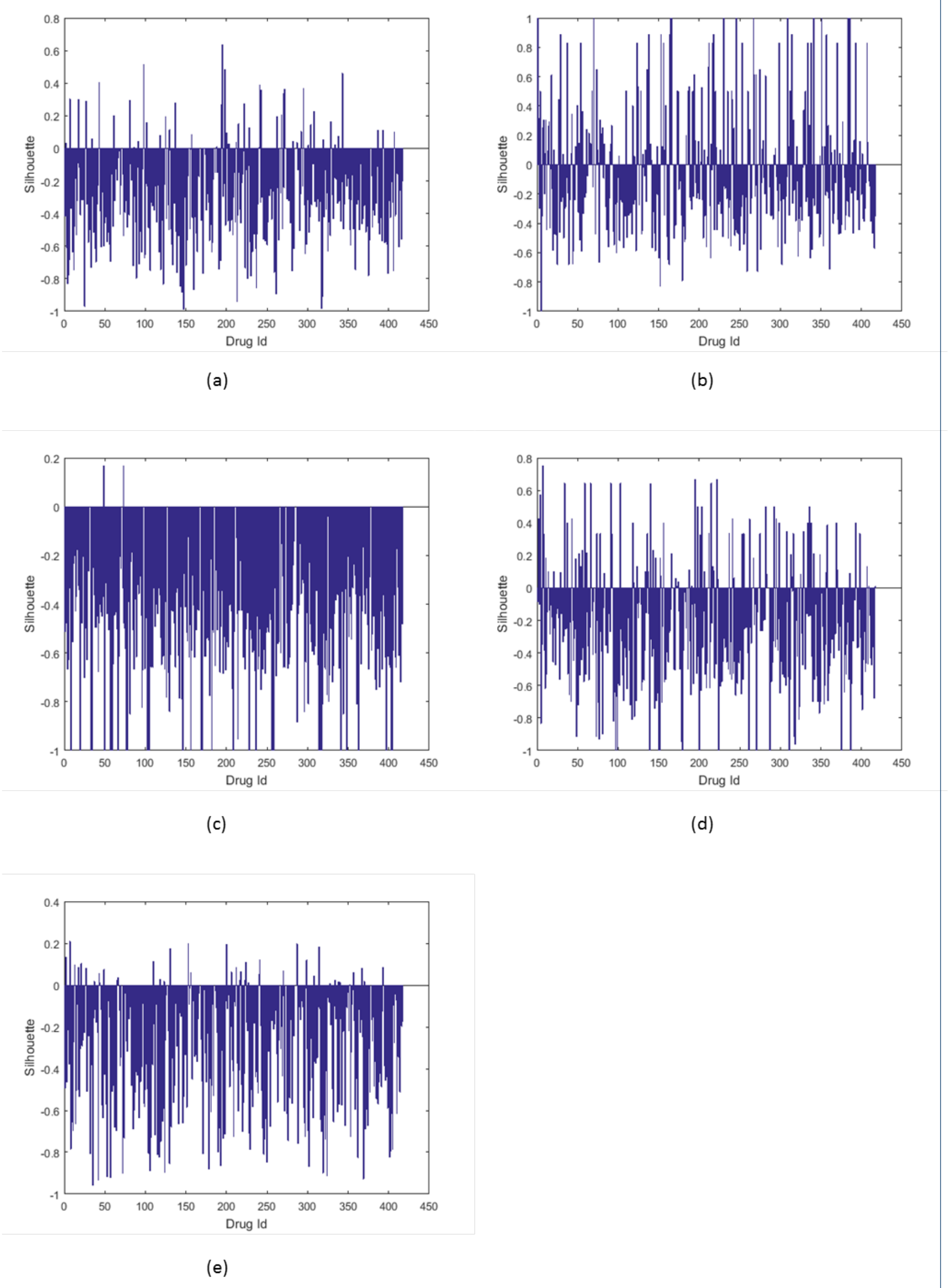

Figure 1: Silhouette comparison for different drug profiles ((a) chemical, (b) disease, (c) gene, (d) protein, and (e) side effect) based on ATC classification.

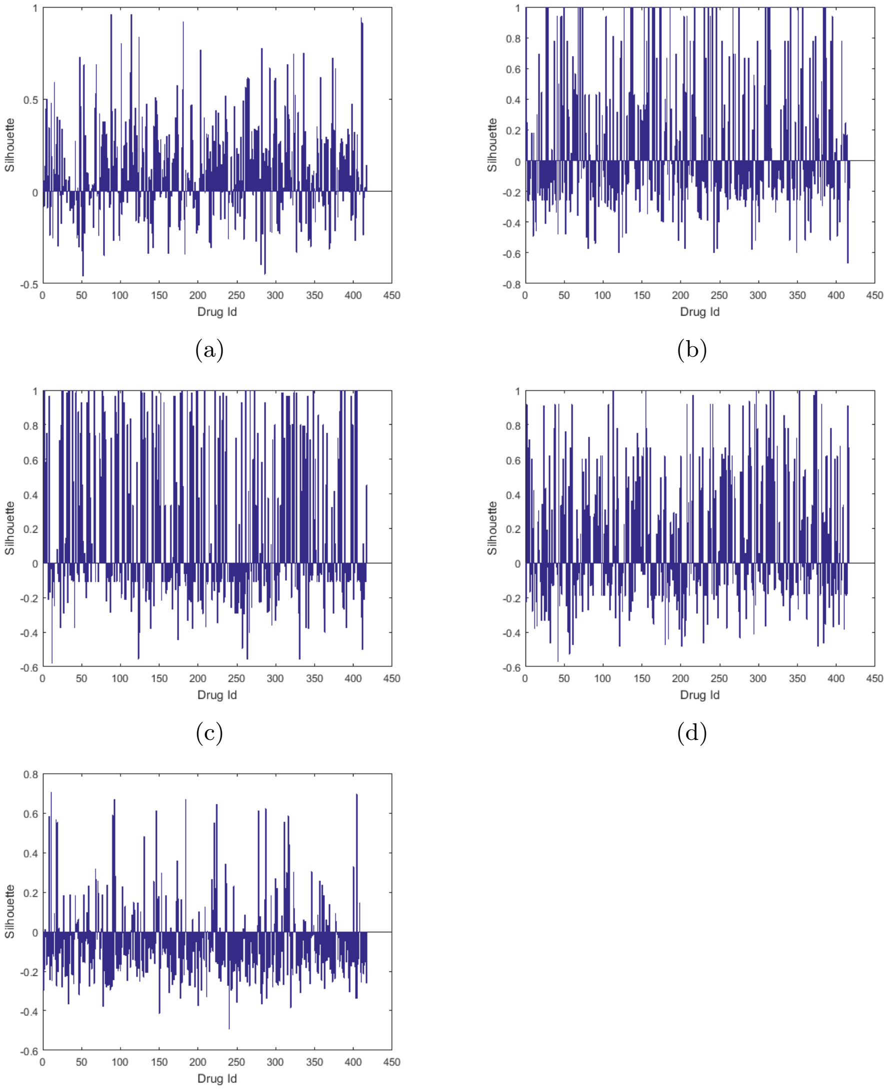

(a) (b) (c) (d) (e)

Figure 2: Silhouette comparison for different drug profiles ((a) chemical, (b) disease, (c) gene, (d) protein, and (e) side effects) based on the clusters generated using GSOM
