# Supplementary material for: A two-tiered unsupervised clustering approach for drug repositioning through heterogeneous data integration
Source: BMC Bioinformatics. 2018 Apr 11;19:129. doi: 10.1186/s12859-018-2123-4 (PMC5896044; doi:10.1186/s12859-018-2123-4)
Supplement: Supplementary file 2 — The 26 pairs of drugs which occur together in Drug Clustering Tier 1. These drug pairs occur together in each drug cluster, generated based on individual chemical, disease, protein and side effect profiles. (PDF 59 kb) [file 12859_2018_2123_MOESM2_ESM.pdf]

Drug 1	ATC L2	Drug 2	ATC L2
Alclometasone	D07/S01	amcinonide	D07
Alclometasone	D07/S01	Desoximetasone	D07
Alclometasone	D07/S01	prednicarbate	D07
amcinonide	D07	desoximetasone	D07
amcinonide	D07	prednicarbate	D07
anastrozole	L02	letrozole	L02
ciclesonide	R01/R03	mometasone	R01/R03/D07
Desoximetasone	D07	prednicarbate	D07
Enalapril	C09	ramipril	C09
Enflurane	N01	halothane	N01
Fluphenazine	N05	Thioridazine	
Fluvoxamine	N06	sertraline	N06
Fosinopril	C09	lisinopril	C09
Fosinopril	C09	trandolapril	C09
Fosinopril	C09	lisinopril	C09
Fosinopril	C09	trandolapril	C09
Hydrochlorothiazide	C03	hydroflumethiazide	C03
Hydrochlorothiazide	C03	methyclothiazide	C03
Hydrochlorothiazide	C03	methyclothiazide	C03
irbesartan	C09	valsartan	C09
Lisinopril	C09	trandolapril	C09
Lovastatin	C10	pravastatin	C10
Lovastatin	C10	simvastatin	C10
Pentobarbital	N05	secobarbital	N05
Pravastatin	C10	simvastatin	C10
Tolazamide	A10	tolbutamide	A10/V04
