# Supplementary material for: A two-tiered unsupervised clustering approach for drug repositioning through heterogeneous data integration
Source: BMC Bioinformatics. 2018 Apr 11;19:129. doi: 10.1186/s12859-018-2123-4 (PMC5896044; doi:10.1186/s12859-018-2123-4)
Supplement: Supplementary file 3 — The repositioning candidates identified consistently by at least two clustering algorithms. This includes the complete list of consistent repositioning candidates, algorithm names and their confidence measures. (PDF 104 kb) [file 12859_2018_2123_MOESM3_ESM.pdf]

Drug Name	Cluster ID	Old ATC	New ATC	Confidence	Algorithm
Allopurinol	5	M04	L01	0.15	CL1
Allopurinol	417	M04	L01	0.10	MCL
Amantadine	51	N04	N05	0.80	CL1
Amantadine	37	N04	N05	0.23	CL1
Amifostine	5	V03	L01	0.15	CL1
Amifostine	417	V03	L01	0.10	MCL
Amiloride	32	C03	C09	0.33	CL1
Amiloride	53	C03	C09	0.33	MCODE
Aminoglutethimide	52	L02	N04	0.25	MCODE
Aminoglutethimide	1	L02	A10	0.21	GSOM
Aminoglutethimide	30	L02	N03	0.17	CL1
Aminoglutethimide	30	L02	N04	0.17	CL1
Aminoglutethimide	16	L02	A10	0.12	CL1
Aminoglutethimide	16	L02	N03	0.12	CL1
Amiodarone	56	C01	N01	0.25	GSOM
Amiodarone	23	C01	N01	0.15	CL1
Amlodipine	403	C08	C09	0.85	MCL
Amlodipine	11	C08	C09	0.70	CL1
Apraclonidine	5	S01	L01	0.15	CL1
Apraclonidine	417	S01	L01	0.10	MCL
Aripiprazole	45	N05	N06	0.47	CL1
Aripiprazole	31	N05	N06	0.40	CL1
Aripiprazole	60	N05	A03	0.20	CL1
Aripiprazole	60	N05	C03	0.20	CL1
Aripiprazole	60	N05	G04	0.20	CL1
Aripiprazole	53	N05	A03	0.17	CL1
Aripiprazole	53	N05	C03	0.17	CL1
Aripiprazole	53	N05	G04	0.17	CL1
Atorvastatin	3	C10	N05	0.27	CL1
Atorvastatin	58	C10	N05	0.15	CL1
Atovaquone	24	P01	L01	0.25	MCODE
Atovaquone	5	P01	L01	0.15	CL1
Atropine	48	A03	N04	0.50	CL1
Atropine	48	S01	N04	0.50	CL1
Atropine	393	A03	N04	0.50	MCL
Atropine	20	A03	N04	0.46	GSOM
Atropine	20	S01	N04	0.46	GSOM
Azelastine	33	S01	A10	0.25	MCODE
Azelastine	28	S01	A10	0.13	CL1
Brinzolamide	48	S01	L02	0.67	MCODE
Brinzolamide	9	S01	L02	0.17	CL1
Bromocriptine	61	N04	N05	0.27	CL1
Bromocriptine	0	N04	N05	0.07	MCODE
Bumetanide	28	C03	A10	0.13	CL1
Bumetanide	28	C03	N03	0.13	CL1
Bumetanide	16	C03	A10	0.12	CL1
Bumetanide	16	C03	N03	0.12	CL1
Caffeine	86	N06	C04	0.20	GSOM
Caffeine	86	N06	N07	0.20	GSOM

Caffeine	86	N06	S01	0.20	GSOM
Caffeine	8	N06	C04	0.11	MCODE
Caffeine	8	N06	N07	0.11	MCODE
Caffeine	8	N06	S01	0.11	MCODE
Capecitabine	3	L01	N05	0.27	CL1
Capecitabine	0	L01	N05	0.07	MCODE
Carbachol	86	N07	C04	0.20	GSOM
Carbachol	86	N07	N06	0.20	GSOM
Carbachol	86	S01	C04	0.20	GSOM
Carbachol	86	S01	N06	0.20	GSOM
Carbachol	8	N07	C04	0.11	MCODE
Carbachol	8	N07	N06	0.11	MCODE
Carbachol	8	S01	C04	0.11	MCODE
Carbachol	8	S01	N06	0.11	MCODE
Carbamazepine	46	N03	N05	0.43	GSOM
Carbamazepine	46	N03	N06	0.43	GSOM
Carbamazepine	25	N03	N06	0.27	CL1
Carbamazepine	42	N03	N05	0.23	CL1
Carbamazepine	20	N03	N05	0.20	MCODE
Carbamazepine	20	N03	N06	0.20	MCODE
Cefazolin	8	J01	L01	0.22	CL1
Cefazolin	24	J01	L01	0.12	CL1
Chloramphenicol	5	G01	L01	0.18	MCODE
Chloramphenicol	5	J01	L01	0.18	MCODE
Chloramphenicol	5	S01	L01	0.18	MCODE
Chloramphenicol	5	S03	L01	0.18	MCODE
Chloramphenicol	24	G01	L01	0.12	CL1
Chloramphenicol	24	S01	L01	0.12	CL1
Chloramphenicol	24	S03	L01	0.12	CL1
Chloramphenicol	417	G01	L01	0.10	MCL
Chloramphenicol	417	J01	L01	0.10	MCL
Chlorzoxazone	88	M03	P03	0.40	GSOM
Chlorzoxazone	8	M03	P03	0.11	MCODE
Ciclesonide	33	R01	D07	0.24	CL1
Ciclesonide	412	R01	D07	0.24	MCL
Ciprofloxacin	8	S02	L01	0.22	CL1
Ciprofloxacin	417	S02	L01	0.10	MCL
Clonidine	56	C02	N05	0.62	CL1
Clonidine	56	S01	N05	0.62	CL1
Clonidine	61	C02	N05	0.27	CL1
Clonidine	61	S01	N05	0.27	CL1
Clotrimazole	371	D01	J02	0.20	MCL
Clotrimazole	55	D01	J02	0.18	GSOM
Cyproheptadine	46	R06	N06	0.70	CL1
Cyproheptadine	35	R06	N06	0.59	CL1
Cyproheptadine	4	R06	N06	0.54	CL1
Cyproheptadine	56	R06	N06	0.25	MCODE
Dexmedetomidine	52	N05	N01	0.30	CL1
Dexmedetomidine	366	N05	N01	0.25	MCL
Dexrazoxane	12	V03	L01	0.33	CL1

Dexrazoxane	24	V03	L01	0.33	GSOM
Diazoxide	5	V03	L01	0.15	CL1
Diazoxide	417	C02	L01	0.10	MCL
Diazoxide	13	C02	L01	0.06	MCODE
Diazoxide	13	V03	L01	0.06	MCODE
Didanosine	92	J05	B01	0.33	GSOM
Didanosine	92	J05	L01	0.33	GSOM
Didanosine	5	J05	L01	0.15	CL1
Didanosine	24	J05	L01	0.12	CL1
Didanosine	13	J05	B01	0.06	MCODE
Didanosine	13	J05	L01	0.06	MCODE
Dihydroergotamine	61	N02	N05	0.27	CL1
Dihydroergotamine	42	N02	N05	0.23	CL1
Disulfiram	24	N07	L01	0.12	CL1
Disulfiram	13	N07	L01	0.06	MCODE
Dolasetron	2	A04	A02	0.40	MCODE
Dolasetron	59	A04	L01	0.20	CL1
Dolasetron	40	A04	A02	0.20	GSOM
Dolasetron	24	A04	L01	0.12	CL1
Doxazosin	60	C02	G04	0.20	CL1
Doxazosin	60	C02	N05	0.20	CL1
Doxazosin	58	C02	G04	0.15	CL1
Doxazosin	58	C02	N05	0.15	CL1
Droperidol	28	N05	N01	0.42	GSOM
Droperidol	359	N05	N03	0.40	MCL
Droperidol	40	N05	N01	0.32	CL1
Droperidol	30	N05	N03	0.17	CL1
Entacapone	15	N04	L01	0.17	MCODE
Entacapone	24	N04	L01	0.12	CL1
Eplerenone	28	C03	J01	0.25	MCODE
Eplerenone	41	C03	J01	0.17	GSOM
Ethotoin	1	N03	C03	0.22	CL1
Ethotoin	28	N03	A10	0.13	CL1
Ethotoin	28	N03	C03	0.13	CL1
Ethotoin	16	N03	A10	0.12	CL1
Ezetimibe	373	C10	A02	0.27	MCL
Ezetimibe	22	C10	A02	0.20	CL1
Famotidine	11	A02	C10	0.27	GSOM
Famotidine	22	A02	C10	0.20	CL1
Fenofibrate	373	C10	A02	0.27	MCL
Fenofibrate	22	C10	A02	0.20	CL1
Fulvestrant	23	L02	A10	0.42	GSOM
Fulvestrant	13	L02	A10	0.42	CL1
Furosemide	60	C03	A03	0.20	CL1
Furosemide	60	C03	G04	0.20	CL1
Furosemide	60	C03	N05	0.20	CL1
Furosemide	53	C03	A03	0.17	CL1
Furosemide	53	C03	G04	0.17	CL1
Furosemide	53	C03	N05	0.17	CL1
Gefitinib	92	L01	B01	0.33	GSOM

Gefitinib	92	L01	J05	0.33	GSOM
Gefitinib	13	L01	B01	0.06	MCODE
Gefitinib	13	L01	J05	0.06	MCODE
Glimepiride	33	A10	R06	0.25	MCODE
Glimepiride	28	A10	R06	0.13	CL1
Hexachlorophene	11	D08	C10	0.27	GSOM
Hexachlorophene	22	D08	C10	0.20	CL1
Hydroxocobalamin	52	B03	L02	0.25	MCODE
Hydroxocobalamin	52	V03	L02	0.25	MCODE
Hydroxocobalamin	9	B03	L02	0.17	CL1
Hydroxocobalamin	9	V03	L02	0.17	CL1
Imiquimod	58	D06	S01	0.33	GSOM
Imiquimod	12	D06	S01	0.20	MCODE
Indapamide	1	C03	L01	0.27	MCODE
Indapamide	24	C03	L01	0.12	CL1
Ivermectin	11	D11	C10	0.27	GSOM
Ivermectin	11	P02	C10	0.27	GSOM
Ivermectin	45	D11	C10	0.25	MCODE
Ivermectin	45	P02	C10	0.25	MCODE
Ketorolac	40	S01	N01	0.32	CL1
Ketorolac	52	S01	N01	0.30	CL1
Leflunomide	62	L04	N04	0.33	MCODE
Leflunomide	30	L04	N04	0.17	CL1
Lidocaine	25	C05	N06	0.27	CL1
Lidocaine	25	N01	N06	0.27	CL1
Lidocaine	42	D04	N05	0.23	CL1
Lidocaine	37	D04	N05	0.23	CL1
Lidocaine	41	C05	N06	0.22	MCODE
Lidocaine	41	N01	N06	0.22	MCODE
Mefloquine	3	P01	D11	0.25	MCODE
Mefloquine	15	P01	D11	0.22	GSOM
Metformin	79	A10	L01	0.50	GSOM
Metformin	21	A10	L01	0.33	MCODE
Methadone	64	N07	A03	0.25	GSOM
Methadone	408	N07	A03	0.25	MCL
Metoclopramide	64	A03	N07	0.25	GSOM
Metoclopramide	408	A03	N07	0.25	MCL
Metoclopramide	37	A03	N05	0.23	CL1
Metoclopramide	60	A03	C03	0.20	CL1
Metoclopramide	60	A03	G04	0.20	CL1
Metoclopramide	60	A03	N05	0.20	CL1
Metoclopramide	53	A03	C03	0.17	CL1
Metoclopramide	53	A03	G04	0.17	CL1
Metoclopramide	53	A03	N05	0.17	CL1
Mexiletine	39	C01	N01	0.33	MCODE
Mexiletine	39	C01	N07	0.33	MCODE
Mexiletine	56	C01	N01	0.25	GSOM
Mexiletine	23	C01	N01	0.15	CL1
Mexiletine	23	C01	N07	0.15	CL1
Milrinone	29	C01	R03	0.25	GSOM

Milrinone	59	C01	R03	0.20	CL1
Moexipril	26	C09	C07	0.50	CL1
Moexipril	49	C09	C07	0.33	CL1
Morphine	56	N02	C01	0.25	GSOM
Morphine	56	N02	N01	0.25	GSOM
Morphine	23	N02	C01	0.15	CL1
Morphine	23	N02	N01	0.15	CL1
Mycophenolic Acid	342	L04	N03	0.50	MCL
Mycophenolic Acid	22	L04	N03	0.27	GSOM
Naloxone	56	V03	C01	0.25	GSOM
Naloxone	19	V03	C01	0.17	CL1
Nitric Oxide	38	R07	B01	0.33	GSOM
Nitric Oxide	38	R07	B02	0.33	GSOM
Nitric Oxide	13	R07	B01	0.06	MCODE
Nitric Oxide	13	R07	B02	0.06	MCODE
Olopatadine	87	S01	G03	0.20	GSOM
Olopatadine	12	S01	G03	0.20	MCODE
Orlistat	26	A08	C02	0.17	MCODE
Orlistat	26	A08	M04	0.17	MCODE
Orlistat	60	A08	C02	0.13	GSOM
Orlistat	60	A08	M04	0.13	GSOM
Oxandrolone	86	A14	N07	0.20	GSOM
Oxandrolone	86	A14	S01	0.20	GSOM
Oxandrolone	13	A14	N07	0.06	MCODE
Oxandrolone	13	A14	S01	0.06	MCODE
Oxazepam	22	N05	N03	0.27	GSOM
Oxazepam	15	N05	N03	0.21	CL1
Paricalcitol	3	H05	D11	0.25	MCODE
Paricalcitol	15	H05	D11	0.22	GSOM
Pentazocine	382	N02	N01	0.27	MCL
Pentazocine	63	N02	N01	0.22	GSOM
Phenoxybenzamine	86	C04	N06	0.20	GSOM
Phenoxybenzamine	86	C04	N07	0.20	GSOM
Phenoxybenzamine	86	C04	S01	0.20	GSOM
Phenoxybenzamine	8	C04	N06	0.11	MCODE
Phenoxybenzamine	8	C04	N07	0.11	MCODE
Phenoxybenzamine	8	C04	S01	0.11	MCODE
Procainamide	20	C01	N03	0.20	MCODE
Procainamide	20	C01	N05	0.20	MCODE
Procainamide	30	C01	N03	0.17	CL1
Procainamide	30	C01	N05	0.17	CL1
Procyclidine	85	N04	S01	0.33	GSOM
Procyclidine	13	N04	S01	0.06	MCODE
Ropinirole	52	N04	L02	0.25	MCODE
Ropinirole	30	N04	L02	0.17	CL1
Sildenafil	60	G04	A03	0.20	CL1
Sildenafil	60	G04	C03	0.20	CL1
Sildenafil	60	G04	N05	0.20	CL1
Sildenafil	53	G04	A03	0.17	CL1
Sildenafil	53	G04	C03	0.17	CL1

Sildenafil	53	G04	N05	0.17	CL1
Tetracycline	13	A01	L01	0.31	GSOM
Tetracycline	13	D06	L01	0.31	GSOM
Tetracycline	13	J01	L01	0.31	GSOM
Tetracycline	331	A01	L01	0.14	MCL
Tetracycline	331	D06	L01	0.14	MCL
Tetracycline	331	J01	L01	0.14	MCL
Tetracycline	331	S01	L01	0.14	MCL
Tetracycline	331	S02	L01	0.14	MCL
Tetracycline	331	S03	L01	0.14	MCL
Tetracycline	13	A01	L01	0.06	MCODE
Tetracycline	13	D06	L01	0.06	MCODE
Tetracycline	13	J01	L01	0.06	MCODE
Tetracycline	13	S01	L01	0.06	MCODE
Tetracycline	13	S02	L01	0.06	MCODE
Tetracycline	13	S03	L01	0.06	MCODE
Tizanidine	372	M03	N02	0.25	MCL
Tizanidine	63	M03	N02	0.25	MCODE
