# Supplementary material for: A two-tiered unsupervised clustering approach for drug repositioning through heterogeneous data integration
Source: BMC Bioinformatics. 2018 Apr 11;19:129. doi: 10.1186/s12859-018-2123-4 (PMC5896044; doi:10.1186/s12859-018-2123-4)
Supplement: Supplementary file 4 — The complete prediction list. This includes the complete list of predicted new classifications into ATC therapeutic class and their confidence measures. (PDF 892 kb) [file 12859_2018_2123_MOESM4_ESM.pdf]

Drug name	Cluster ID	Old ATC name	New ATC name	Confidence	Algorithm
Amlodipine	403	C08	C09	0.846154	mcl
Chlorthalidone	2		C03	0.833333	CL1
Amantadine	51	N04	N05	0.8	CL1
Thioridazine	51		N05	0.8	CL1
Hydroxyzine	30	N05	C09	0.75	mcode
Cyproheptadin	46	R06	N06	0.7	CL1
Amlodipine	11	C08	C09	0.695652	CL1
Carvedilol	11	C07	C09	0.695652	CL1
Cetirizine	11	R06	C09	0.695652	CL1
Acitretin	414	D05	D10	0.666667	mcl
Brinzolamide	48	S01	L02	0.666667	mcode
Orphenadrine	392		R06	0.642857	mcl
Clonidine	56	C02	N05	0.615385	CL1
Clonidine	56	N02	N05	0.615385	CL1
Clonidine	56	S01	N05	0.615385	CL1
Thioridazine	56		N05	0.615385	CL1
Dofetilide	399	C01	L02	0.6	mcl
Cyproheptadin	35	R06	N06	0.590909	CL1
Guanfacine	35	C02	N06	0.590909	CL1
Dipivefrin	44	S01	N05	0.571429	CL1
Indomethacin	7		M01	0.571429	mcode
Nicardipine	57	C08	N06	0.571429	CL1
Cyproheptadin	4	R06	N06	0.541667	CL1
Methadone	4	N07	N06	0.541667	CL1
Arsenic Trioxid	4	L01	P01	0.5	mcode
Atropine	48	A03	N04	0.5	CL1
Atropine	48	S01	N04	0.5	CL1
Atropine	393	A03	N04	0.5	mcl
Dacarbazine	79	L01	A10	0.5	gsom
Hexachlorophe	350	D08	D05	0.5	mcl
Isocarboxazid	50	N06	N05	0.5	mcode
Levetiracetam	346	N03	L01	0.5	mcl
Lithium	6	N05	N06	0.5	gsom
Mercaptopurin	4	L01	P01	0.5	mcode
Metformin	79	A10	L01	0.5	gsom
Moexipril	26	C09	C07	0.5	CL1
Mycophenolic	342	L04	N03	0.5	mcl
Phenytoin	66	N03	C01	0.5	gsom
Tazarotene	350	D05	D08	0.5	mcl
Tolterodine	59	G04	C01	0.5	gsom
Topotecan	346	L01	N03	0.5	mcl
Zonisamide	342	N03	L04	0.5	mcl
Aspirin	6		M01	0.470588	CL1
Indomethacin	6		M01	0.470588	CL1
Aripiprazole	45	N05	N06	0.466667	CL1
Ofloxacin	45	S01	N06	0.466667	CL1
Atropine	20	A03	N04	0.461538	gsom
Atropine	20	S01	N04	0.461538	gsom
Indomethacin	380		M01	0.457143	mcl

Epinephrine	17	A01	C07	0.454545	CL1
Epinephrine	17	B02	C07	0.454545	CL1
Epinephrine	17	R01	C07	0.454545	CL1
Epinephrine	17	R03	C07	0.454545	CL1
Epinephrine	17	S01	C07	0.454545	CL1
Carbamazepine	46	N03	N05	0.428571	gsom
Carbamazepine	46	N03	N06	0.428571	gsom
Orphenadrine	36		R06	0.428571	CL1
Sertraline	38	N06	A10	0.428571	mcode
Droperidol	28	N05	N01	0.416667	gsom
Fulvestrant	23	L02	A10	0.416667	gsom
Fulvestrant	13	L02	A10	0.416667	CL1
Bicalutamide	10	L02	C10	0.411765	CL1
Aripiprazole	31	N05	N06	0.4	CL1
Bimatoprost	55	S01	L02	0.4	mcode
Cefdinir	42	J01	M03	0.4	gsom
Chlorzoxazone	88	M03	P03	0.4	gsom
Dantrolene	47	M03	B01	0.4	mcode
Digoxin	2	C01	A02	0.4	mcode
Dolasetron	2	A04	A02	0.4	mcode
Donepezil	2	N06	A02	0.4	mcode
Droperidol	359	N05	N03	0.4	mcl
Entacapone	42	N04	M03	0.4	gsom
Hydroxyurea	2		A02	0.4	mcode
Lindane	34	P03	C08	0.4	mcode
Mephenytoin	18	N03	P01	0.4	gsom
Methazolamide	34	S01	C08	0.4	mcode
Metipranolol	42	S01	C07	0.4	mcode
Procaine	31	C05	N06	0.4	CL1
Procaine	61	N01	N06	0.4	mcode
Spironolactone	47	C03	B01	0.4	mcode
Tadalafil	49	G04	C09	0.4	mcode
Albendazole	14	P02	A02	0.333333	mcode
Albendazole	14	P02	D01	0.333333	mcode
Amifostine	11	V03	H02	0.333333	mcode
Amifostine	11	V03	S01	0.333333	mcode
Amiloride	32	C03	C09	0.333333	CL1
Amiloride	53	C03	C09	0.333333	mcode
Amiloride	53	C03	N03	0.333333	mcode
Amiodarone	49	C01	C07	0.333333	CL1
Aspirin	14		A02	0.333333	mcode
Aspirin	14		D01	0.333333	mcode
Aspirin	14		P02	0.333333	mcode
Buspirone	386	N05	N04	0.333333	mcl
Buspirone	386	N05	N06	0.333333	mcl
Captopril	53	C09	C03	0.333333	mcode
Captopril	53	C09	N03	0.333333	mcode
Desloratadine	50	R06	R03	0.333333	CL1
Dexrazoxane	12	V03	L01	0.333333	CL1
Eprosartan	62	C09	L04	0.333333	mcode

Eprosartan	62	C09	N04	0.333333	mcode
Fenoprofen	32	M01	C09	0.333333	CL1
Fludrocortisone	11	H02	S01	0.333333	mcode
Fludrocortisone	11	H02	V03	0.333333	mcode
Fulvestrant	54	L02	A02	0.333333	mcode
Fulvestrant	54	L02	N03	0.333333	mcode
Glycopyrrolate	21		A10	0.333333	mcode
Glycopyrrolate	21		D11	0.333333	mcode
Glycopyrrolate	21		L01	0.333333	mcode
Lamotrigine	54	N03	A02	0.333333	mcode
Lamotrigine	54	N03	L02	0.333333	mcode
Leflunomide	62	L04	C09	0.333333	mcode
Leflunomide	62	L04	N04	0.333333	mcode
Levobunolol	51	S01	C07	0.333333	mcode
Levobunolol	51	S01	R06	0.333333	mcode
Loratadine	51	R06	C07	0.333333	mcode
Loratadine	51	R06	S01	0.333333	mcode
Mebendazole	16	P02	L02	0.333333	mcode
Mebendazole	16	P02	N01	0.333333	mcode
Metformin	21	A10	D11	0.333333	mcode
Metformin	21	A10	L01	0.333333	mcode
Mexiletine	39	C01	N01	0.333333	mcode
Mexiletine	39	C01	N07	0.333333	mcode
Moexipril	49	C09	C01	0.333333	CL1
Moexipril	49	C09	C07	0.333333	CL1
Naltrexone	39	N07	C01	0.333333	mcode
Naltrexone	39	N07	N01	0.333333	mcode
Nilutamide	16	L02	N01	0.333333	mcode
Nilutamide	16	L02	P02	0.333333	mcode
Nizatidine	54	A02	L02	0.333333	mcode
Nizatidine	54	A02	N03	0.333333	mcode
Norfloxacin	32	J01	C09	0.333333	CL1
Norfloxacin	32	S01	C09	0.333333	CL1
Penbutolol	51	C07	R06	0.333333	mcode
Penbutolol	51	C07	S01	0.333333	mcode
Pimecrolimus	21	D11	A10	0.333333	mcode
Pimecrolimus	21	D11	L01	0.333333	mcode
Pramipexole	386	N04	N05	0.333333	mcl
Pramipexole	386	N04	N06	0.333333	mcl
Remifentanil	16	N01	L02	0.333333	mcode
Remifentanil	16	N01	P02	0.333333	mcode
Selegiline	62	N04	C09	0.333333	mcode
Selegiline	62	N04	L04	0.333333	mcode
Sufentanil	39	N01	C01	0.333333	mcode
Sufentanil	39	N01	N07	0.333333	mcode
Trazodone	386	N06	N04	0.333333	mcl
Trazodone	386	N06	N05	0.333333	mcl
Tropicamide	11	S01	H02	0.333333	mcode
Tropicamide	11	S01	V03	0.333333	mcode
Valproic Acid	53	N03	C03	0.333333	mcode

Valproic Acid	53	N03	C09	0.333333	mcode
Acarbose	17	A10	L01	0.333333	gsom
Acarbose	17	A10	M04	0.333333	gsom
Argatroban	38	B01	B02	0.333333	gsom
Argatroban	38	B01	R07	0.333333	gsom
Capecitabine	17	L01	A10	0.333333	gsom
Capecitabine	17	L01	M04	0.333333	gsom
Carvedilol	34	C07	N03	0.333333	gsom
Carvedilol	34	C07	N06	0.333333	gsom
Cefixime	80	J01	B01	0.333333	gsom
Cefixime	80	J01	R03	0.333333	gsom
Dexrazoxane	24	V03	L01	0.333333	gsom
Didanosine	92	J05	B01	0.333333	gsom
Didanosine	92	J05	L01	0.333333	gsom
Emedastine	58	S01	N07	0.333333	gsom
Galantamine	34	N06	C07	0.333333	gsom
Galantamine	34	N06	N03	0.333333	gsom
Gefitinib	92	L01	B01	0.333333	gsom
Gefitinib	92	L01	J05	0.333333	gsom
Imiquimod	58	D06	N07	0.333333	gsom
Imiquimod	58	D06	S01	0.333333	gsom
Levocabastine	85	R01	N04	0.333333	gsom
Levocabastine	85	S01	N04	0.333333	gsom
Nitric Oxide	38	R07	B01	0.333333	gsom
Nitric Oxide	38	R07	B02	0.333333	gsom
Procyclidine	85	N04	R01	0.333333	gsom
Procyclidine	85	N04	S01	0.333333	gsom
Sulfinpyrazone	17	M04	A10	0.333333	gsom
Sulfinpyrazone	17	M04	L01	0.333333	gsom
Ticlopidine	92	B01	J05	0.333333	gsom
Ticlopidine	92	B01	L01	0.333333	gsom
Tranexamic Aci	38	B02	B01	0.333333	gsom
Tranexamic Aci	38	B02	R07	0.333333	gsom
Valproic Acid	34	N03	C07	0.333333	gsom
Valproic Acid	34	N03	N06	0.333333	gsom
Warfarin	80	B01	J01	0.333333	gsom
Warfarin	80	B01	R03	0.333333	gsom
Zafirlukast	80	R03	B01	0.333333	gsom
Zafirlukast	80	R03	J01	0.333333	gsom
Dopamine	40	C01	N01	0.323529	CL1
Droperidol	40	N05	N01	0.323529	CL1
Ketorolac	40	S01	N01	0.323529	CL1
Leflunomide	13	L04	L01	0.307692	gsom
Tetracycline	13	A01	L01	0.307692	gsom
Tetracycline	13	D06	L01	0.307692	gsom
Tetracycline	13	J01	L01	0.307692	gsom
Brimonidine	54	S01	C02	0.3	CL1
Aripiprazole	52	N05	N01	0.296296	CL1
Dexmedetomic	52	N05	N01	0.296296	CL1
Ketorolac	52	M01	N01	0.296296	CL1

Ketorolac	52	S01	N01	0.296296	CL1
Azelaic Acid	22	D10	N03	0.272727	gsom
Famotidine	11	A02	C10	0.272727	gsom
Hexachlorophe	11	D08	C10	0.272727	gsom
Ivermectin	11	D11	C10	0.272727	gsom
Ivermectin	11	P02	C10	0.272727	gsom
Mycophenolic /	22	L04	N03	0.272727	gsom
Oxazepam	22	N05	N03	0.272727	gsom
Atorvastatin	3	C10	N05	0.272727	CL1
Bromocriptine	55	G02	N02	0.272727	CL1
Bromocriptine	55	N04	N02	0.272727	CL1
Bromocriptine	61	G02	N05	0.272727	CL1
Bromocriptine	61	N04	N05	0.272727	CL1
Capecitabine	3	L01	N05	0.272727	CL1
Carbamazepine	382	N03	N01	0.272727	mcl
Clonidine	61	C02	N05	0.272727	CL1
Clonidine	61	S01	N05	0.272727	CL1
Dihydroergotar	61	N02	N05	0.272727	CL1
Ezetimibe	373	C10	A02	0.272727	mcl
Fenofibrate	373	C10	A02	0.272727	mcl
Fluvastatin	3	C10	N05	0.272727	CL1
Gemfibrozil	373	C10	A02	0.272727	mcl
Isradipine	3	C08	N05	0.272727	CL1
Mycophenolic /	3	L04	N05	0.272727	CL1
Pentazocine	382	N02	N01	0.272727	mcl
Bicalutamide	25	L02	N06	0.269231	CL1
Carbamazepine	25	N03	N06	0.269231	CL1
Finasteride	25	G04	N06	0.269231	CL1
Flutamide	25	L02	N06	0.269231	CL1
Lidocaine	25	C05	N06	0.269231	CL1
Lidocaine	25	D04	N06	0.269231	CL1
Lidocaine	25	N01	N06	0.269231	CL1
Indapamide	1	C03	L01	0.266667	mcode
Minoxidil	1	D11	L01	0.266667	mcode
Nicotine	1	N07	L01	0.266667	mcode
Adenosine	17	C01	R06	0.25	mcode
Amantadine	408	N04	A03	0.25	mcl
Amantadine	408	N04	A04	0.25	mcl
Amantadine	408	N04	N07	0.25	mcl
Aminoglutethir	52	L02	B03	0.25	mcode
Aminoglutethir	52	L02	N04	0.25	mcode
Aminoglutethir	52	L02	V03	0.25	mcode
Amiodarone	56	C01	N01	0.25	gsom
Atovaquone	24	P01	C01	0.25	mcode
Atovaquone	24	P01	C08	0.25	mcode
Atovaquone	24	P01	L01	0.25	mcode
Atropine	56	A03	N06	0.25	mcode
Atropine	56	A03	R06	0.25	mcode
Atropine	56	S01	N06	0.25	mcode
Atropine	56	S01	R06	0.25	mcode

Azelastine	33	R01	A10	0.25	mcode
Azelastine	33	R06	A10	0.25	mcode
Azelastine	33	S01	A10	0.25	mcode
Baclofen	28	M03	C03	0.25	mcode
Baclofen	28	M03	J01	0.25	mcode
Baclofen	28	M03	N02	0.25	mcode
Carteolol	59	C07	A10	0.25	mcode
Carteolol	59	C07	B01	0.25	mcode
Carteolol	59	S01	A10	0.25	mcode
Carteolol	59	S01	B01	0.25	mcode
Cefixime	28	J01	C03	0.25	mcode
Cefixime	28	J01	M03	0.25	mcode
Cefixime	28	J01	N02	0.25	mcode
Clonidine	21	C02	C07	0.25	gsom
Clonidine	21	N02	C07	0.25	gsom
Clonidine	372	C02	M03	0.25	mcl
Clonidine	372	N02	M03	0.25	mcl
Clonidine	372	S01	M03	0.25	mcl
Cyproheptadin	56	R06	A03	0.25	mcode
Cyproheptadin	56	R06	N06	0.25	mcode
Cyproheptadin	56	R06	S01	0.25	mcode
Daunorubicin	24	L01	C01	0.25	mcode
Daunorubicin	24	L01	C08	0.25	mcode
Daunorubicin	24	L01	P01	0.25	mcode
Dexmedetomic	366	N05	C05	0.25	mcl
Dexmedetomic	366	N05	N01	0.25	mcl
Dexmedetomic	366	N05	S01	0.25	mcl
Dihydroergotai	63	N02	D11	0.25	mcode
Dihydroergotai	63	N02	G04	0.25	mcode
Dihydroergotai	63	N02	M03	0.25	mcode
Disopyramide	21	C01	C07	0.25	gsom
Dopamine	29	C01	R03	0.25	gsom
Doxazosin	21	C02	C07	0.25	gsom
Eplerenone	28	C03	J01	0.25	mcode
Eplerenone	28	C03	M03	0.25	mcode
Eplerenone	28	C03	N02	0.25	mcode
Ergotamine	29	N02	C01	0.25	gsom
Ergotamine	29	N02	R03	0.25	gsom
Ethionamide	39	J04	C01	0.25	CL1
Ethionamide	45	J04	C10	0.25	mcode
Ethionamide	45	J04	D11	0.25	mcode
Ethionamide	45	J04	P02	0.25	mcode
Ezetimibe	45	C10	D11	0.25	mcode
Ezetimibe	45	C10	J04	0.25	mcode
Ezetimibe	45	C10	P02	0.25	mcode
Finasteride	63	D11	M03	0.25	mcode
Finasteride	63	D11	N02	0.25	mcode
Finasteride	63	G04	M03	0.25	mcode
Finasteride	63	G04	N02	0.25	mcode
Glimepiride	33	A10	R01	0.25	mcode

Glimepiride	33	A10	R06	0.25	mcode
Glimepiride	33	A10	S01	0.25	mcode
Hydroxocobala	52	B03	L02	0.25	mcode
Hydroxocobala	52	B03	N04	0.25	mcode
Hydroxocobala	52	V03	L02	0.25	mcode
Hydroxocobala	52	V03	N04	0.25	mcode
Iloprost	59	B01	A10	0.25	mcode
Iloprost	59	B01	C07	0.25	mcode
Iloprost	59	B01	S01	0.25	mcode
Imatinib	39	L01	C01	0.25	CL1
Imatinib	39	L01	J04	0.25	CL1
Isoflurane	29	N01	C01	0.25	gsom
Isoflurane	29	N01	R03	0.25	gsom
Isoniazid	39	J04	C01	0.25	CL1
Ivermectin	45	D11	C10	0.25	mcode
Ivermectin	45	D11	J04	0.25	mcode
Ivermectin	45	P02	C10	0.25	mcode
Ivermectin	45	P02	J04	0.25	mcode
Labetalol	47	C07	M04	0.25	CL1
Labetalol	47	C07	N01	0.25	CL1
Labetalol	47	C07	N05	0.25	CL1
Losartan	39	C09	C01	0.25	CL1
Losartan	39	C09	J04	0.25	CL1
Mefloquine	3	P01	D11	0.25	mcode
Mefloquine	3	P01	H05	0.25	mcode
Mefloquine	3	P01	L04	0.25	mcode
Megestrol	54	G03	A07	0.25	gsom
Megestrol	54	G03	H02	0.25	gsom
Megestrol	54	L02	A07	0.25	gsom
Megestrol	54	L02	H02	0.25	gsom
Methadone	64	N07	A03	0.25	gsom
Methadone	64	N07	D04	0.25	gsom
Methadone	64	N07	R06	0.25	gsom
Methadone	408	N07	A03	0.25	mcl
Methadone	408	N07	A04	0.25	mcl
Methadone	408	N07	N04	0.25	mcl
Metoclopramic	64	A03	D04	0.25	gsom
Metoclopramic	64	A03	N07	0.25	gsom
Metoclopramic	64	A03	R06	0.25	gsom
Metoclopramic	408	A03	A04	0.25	mcl
Metoclopramic	408	A03	N04	0.25	mcl
Metoclopramic	408	A03	N07	0.25	mcl
Mexiletine	56	C01	N01	0.25	gsom
Midodrine	24	C01	C08	0.25	mcode
Midodrine	24	C01	L01	0.25	mcode
Midodrine	24	C01	P01	0.25	mcode
Milrinone	29	C01	R03	0.25	gsom
Morphine	56	N02	C01	0.25	gsom
Morphine	56	N02	N01	0.25	gsom
Naloxone	56	V03	C01	0.25	gsom

Naloxone	56	V03	N01	0.25	gsom
Nimodipine	24	C08	C01	0.25	mcode
Nimodipine	24	C08	L01	0.25	mcode
Nimodipine	24	C08	P01	0.25	mcode
Ondansetron	408	A04	A03	0.25	mcl
Ondansetron	408	A04	N04	0.25	mcl
Ondansetron	408	A04	N07	0.25	mcl
Orphenadrine	33		A10	0.25	mcode
Orphenadrine	33		R01	0.25	mcode
Orphenadrine	33		R06	0.25	mcode
Orphenadrine	33		S01	0.25	mcode
Paricalcitol	3	H05	D11	0.25	mcode
Paricalcitol	3	H05	L04	0.25	mcode
Paricalcitol	3	H05	P01	0.25	mcode
Phenelzine	56	N06	A03	0.25	mcode
Phenelzine	56	N06	R06	0.25	mcode
Phenelzine	56	N06	S01	0.25	mcode
Pioglitazone	59	A10	B01	0.25	mcode
Pioglitazone	59	A10	C07	0.25	mcode
Pioglitazone	59	A10	S01	0.25	mcode
Prednisone	54	A07	G03	0.25	gsom
Prednisone	54	A07	L02	0.25	gsom
Prednisone	54	H02	G03	0.25	gsom
Prednisone	54	H02	L02	0.25	gsom
Procaine	366	C05	N05	0.25	mcl
Procaine	366	N01	N05	0.25	mcl
Procaine	366	S01	N05	0.25	mcl
Promethazine	64	D04	A03	0.25	gsom
Promethazine	64	D04	N07	0.25	gsom
Promethazine	64	R06	A03	0.25	gsom
Promethazine	64	R06	N07	0.25	gsom
Quetiapine	47	N05	C07	0.25	CL1
Quetiapine	47	N05	M04	0.25	CL1
Quetiapine	47	N05	N01	0.25	CL1
Ropinirole	52	N04	B03	0.25	mcode
Ropinirole	52	N04	L02	0.25	mcode
Ropinirole	52	N04	V03	0.25	mcode
Sufentanil	47	N01	C07	0.25	CL1
Sufentanil	47	N01	M04	0.25	CL1
Sufentanil	47	N01	N05	0.25	CL1
Sulfinpyrazone	47	M04	C07	0.25	CL1
Sulfinpyrazone	47	M04	N01	0.25	CL1
Sulfinpyrazone	47	M04	N05	0.25	CL1
Sumatriptan	28	N02	C03	0.25	mcode
Sumatriptan	28	N02	J01	0.25	mcode
Sumatriptan	28	N02	M03	0.25	mcode
Tacrolimus	3	D11	H05	0.25	mcode
Tacrolimus	3	D11	P01	0.25	mcode
Tacrolimus	3	L04	H05	0.25	mcode
Tacrolimus	3	L04	P01	0.25	mcode

Tizanidine	372	M03	C02	0.25	mcl
Tizanidine	372	M03	N02	0.25	mcl
Tizanidine	372	M03	S01	0.25	mcl
Tizanidine	63	M03	D11	0.25	mcode
Tizanidine	63	M03	G04	0.25	mcode
Tizanidine	63	M03	N02	0.25	mcode
Ciclesonide	33	R01	D07	0.237288	CL1
Ciclesonide	412	R01	D07	0.237288	mcl
Carbamazepine	42	N03	N05	0.230769	CL1
Ciprofloxacin	29	S01	M01	0.230769	mcode
Ciprofloxacin	29	S02	M01	0.230769	mcode
Ciprofloxacin	29	S03	M01	0.230769	mcode
Desipramine	42	N06	N05	0.230769	CL1
Dihydroergotar	42	N02	N05	0.230769	CL1
Finasteride	42	G04	N05	0.230769	CL1
Lidocaine	42	C01	N05	0.230769	CL1
Lidocaine	42	C05	N05	0.230769	CL1
Lidocaine	42	D04	N05	0.230769	CL1
Amantadine	37	N04	N05	0.227273	CL1
Ketamine	37	N01	N05	0.227273	CL1
Lidocaine	37	D04	N05	0.227273	CL1
Lidocaine	37	N01	N05	0.227273	CL1
Lidocaine	37	S01	N05	0.227273	CL1
Lidocaine	37	S02	N05	0.227273	CL1
Methadone	37	N07	N05	0.227273	CL1
Metoclopramic	37	A03	N05	0.227273	CL1
Pentazocine	37	N02	N05	0.227273	CL1
Acarbose	1	A10	C03	0.222222	CL1
Aspirin	26		D07	0.222222	gsom
Aspirin	26		R01	0.222222	gsom
Aspirin	26		R03	0.222222	gsom
Cefazolin	8	J01	L01	0.222222	CL1
Ciprofloxacin	8	J01	L01	0.222222	CL1
Ciprofloxacin	8	S01	L01	0.222222	CL1
Ciprofloxacin	8	S02	L01	0.222222	CL1
Ethotoin	1	N03	C03	0.222222	CL1
Ketoconazole	1	D01	C03	0.222222	CL1
Ketoconazole	1	G01	C03	0.222222	CL1
Ketoconazole	1	J02	C03	0.222222	CL1
Lidocaine	41	C01	N06	0.222222	mcode
Lidocaine	41	C05	N06	0.222222	mcode
Lidocaine	41	N01	N06	0.222222	mcode
Lidocaine	41	R02	N06	0.222222	mcode
Lidocaine	41	S01	N06	0.222222	mcode
Loratadine	1	R06	C03	0.222222	CL1
Penbutolol	1	C07	C03	0.222222	CL1
Apraclonidine	61	S01	V03	0.222222	gsom
Aspirin	26		D07	0.222222	gsom
Aspirin	26		R01	0.222222	gsom
Aspirin	26		R03	0.222222	gsom

Atomoxetine	61	N06	V03	0.222222	gsom
Bosentan	15	C02	D11	0.222222	gsom
Flunisolide	26	R01	D07	0.222222	gsom
Fluorometholo	26	D07	R01	0.222222	gsom
Fluorometholo	26	D07	R03	0.222222	gsom
Mefloquine	15	P01	D11	0.222222	gsom
Midodrine	61	C01	V03	0.222222	gsom
Nalbuphine	61	N02	V03	0.222222	gsom
Paricalcitol	15	H05	D11	0.222222	gsom
Pentazocine	63	N02	N01	0.222222	gsom
Ropivacaine	15	N01	D11	0.222222	gsom
Adapalene	15	D10	N03	0.214286	CL1
Modafinil	15	N06	N03	0.214286	CL1
Oxazepam	15	N05	N03	0.214286	CL1
Acetazolamide	1	S01	A10	0.214286	gsom
Aminoglutethir	1	L02	A10	0.214286	gsom
Azathioprine	1	L04	A10	0.214286	gsom
Cimetidine	1	A02	A10	0.214286	gsom
Griseofulvin	1	D01	A10	0.214286	gsom
Hydroxychloro	1	P01	A10	0.214286	gsom
Isoniazid	1	J04	A10	0.214286	gsom
Probenecid	1	M04	A10	0.214286	gsom
Argatroban	20	B01	C01	0.2	mcode
Argatroban	20	B01	N03	0.2	mcode
Argatroban	20	B01	N05	0.2	mcode
Argatroban	20	B01	N06	0.2	mcode
Aripiprazole	60	N05	A03	0.2	CL1
Aripiprazole	60	N05	C02	0.2	CL1
Aripiprazole	60	N05	C03	0.2	CL1
Aripiprazole	60	N05	G04	0.2	CL1
Bacitracin	21	D06	R06	0.2	CL1
Bacitracin	21	J01	R06	0.2	CL1
Bacitracin	21	R02	R06	0.2	CL1
Biperiden	21	N04	R06	0.2	CL1
Caffeine	21	N06	R06	0.2	CL1
Caffeine	86	N06	A14	0.2	gsom
Caffeine	86	N06	C04	0.2	gsom
Caffeine	86	N06	N07	0.2	gsom
Caffeine	86	N06	S01	0.2	gsom
Carbachol	21	N07	R06	0.2	CL1
Carbachol	86	N07	A14	0.2	gsom
Carbachol	86	N07	C04	0.2	gsom
Carbachol	86	N07	N06	0.2	gsom
Carbachol	86	S01	A14	0.2	gsom
Carbachol	86	S01	C04	0.2	gsom
Carbachol	86	S01	N06	0.2	gsom
Carbamazepine	20	N03	B01	0.2	mcode
Carbamazepine	20	N03	C01	0.2	mcode
Carbamazepine	20	N03	N05	0.2	mcode
Carbamazepine	20	N03	N06	0.2	mcode

Chlorzoxazone	21	M03	R06	0.2	CL1
Clotrimazole	371	D01	J02	0.2	mcl
Clotrimazole	371	G01	J02	0.2	mcl
Diltiazem	40	C08	A02	0.2	gsom
Disulfiram	21	N07	R06	0.2	CL1
Disulfiram	21	P03	R06	0.2	CL1
Docetaxel	40	L01	A02	0.2	gsom
Dofetilide	43	C01	C07	0.2	CL1
Dofetilide	43	C01	G04	0.2	CL1
Dofetilide	43	C01	N03	0.2	CL1
Dofetilide	43	C01	R03	0.2	CL1
Dolasetron	59	A04	L01	0.2	CL1
Dolasetron	59	A04	R03	0.2	CL1
Dolasetron	40	A04	A02	0.2	gsom
Doxazosin	60	C02	A03	0.2	CL1
Doxazosin	60	C02	C03	0.2	CL1
Doxazosin	60	C02	G04	0.2	CL1
Doxazosin	60	C02	N05	0.2	CL1
Emedastine	12	S01	G03	0.2	mcode
Etodolac	59	M01	L01	0.2	CL1
Etodolac	59	M01	R03	0.2	CL1
Ezetimibe	22	C10	A02	0.2	CL1
Famotidine	22	A02	C10	0.2	CL1
Fenofibrate	22	C10	A02	0.2	CL1
Flecainide	12	C01	G03	0.2	mcode
Flecainide	12	C01	S01	0.2	mcode
Formoterol	43	R03	C01	0.2	CL1
Formoterol	43	R03	C07	0.2	CL1
Formoterol	43	R03	G04	0.2	CL1
Formoterol	43	R03	N03	0.2	CL1
Formoterol	59	R03	L01	0.2	CL1
Furosemide	60	C03	A03	0.2	CL1
Furosemide	60	C03	C02	0.2	CL1
Furosemide	60	C03	G04	0.2	CL1
Furosemide	60	C03	N05	0.2	CL1
Griseofulvin	22	D01	A02	0.2	CL1
Griseofulvin	22	D01	C10	0.2	CL1
Hexachlorophe	22	D08	A02	0.2	CL1
Hexachlorophe	22	D08	C10	0.2	CL1
Hydroxychloro	22	P01	A02	0.2	CL1
Hydroxychloro	22	P01	C10	0.2	CL1
Hydroxyzine	21	N05	R06	0.2	CL1
Imatinib	21	L01	R06	0.2	CL1
Imiquimod	12	D06	G03	0.2	mcode
Imiquimod	12	D06	S01	0.2	mcode
Irinotecan	59	L01	R03	0.2	CL1
Isoniazid	22	J04	A02	0.2	CL1
Isoniazid	22	J04	C10	0.2	CL1
Labetalol	43	C07	C01	0.2	CL1
Labetalol	43	C07	G04	0.2	CL1

Labetalol	43	C07	N03	0.2	CL1
Labetalol	43	C07	R03	0.2	CL1
Levetiracetam	43	N03	C01	0.2	CL1
Levetiracetam	43	N03	C07	0.2	CL1
Levetiracetam	43	N03	G04	0.2	CL1
Levetiracetam	43	N03	R03	0.2	CL1
Loperamide	87	A07	C08	0.2	gsom
Loperamide	87	A07	G03	0.2	gsom
Loperamide	87	A07	R01	0.2	gsom
Loperamide	87	A07	S01	0.2	gsom
Lorazepam	20	N05	B01	0.2	mcode
Lorazepam	20	N05	C01	0.2	mcode
Lorazepam	20	N05	N03	0.2	mcode
Lorazepam	20	N05	N06	0.2	mcode
Metoclopramic	60	A03	C02	0.2	CL1
Metoclopramic	60	A03	C03	0.2	CL1
Metoclopramic	60	A03	G04	0.2	CL1
Metoclopramic	60	A03	N05	0.2	CL1
Metolazone	40	C03	A02	0.2	gsom
Mifepristone	87	G03	A07	0.2	gsom
Mifepristone	87	G03	C08	0.2	gsom
Mifepristone	87	G03	R01	0.2	gsom
Mifepristone	87	G03	S01	0.2	gsom
Milrinone	59	C01	L01	0.2	CL1
Milrinone	59	C01	R03	0.2	CL1
Moricizine	20		B01	0.2	mcode
Moricizine	20		C01	0.2	mcode
Moricizine	20		N03	0.2	mcode
Moricizine	20		N05	0.2	mcode
Moricizine	20		N06	0.2	mcode
Nabilone	40	A04	A02	0.2	gsom
Nimodipine	87	C08	A07	0.2	gsom
Nimodipine	87	C08	G03	0.2	gsom
Nimodipine	87	C08	R01	0.2	gsom
Nimodipine	87	C08	S01	0.2	gsom
Olopatadine	87	R01	A07	0.2	gsom
Olopatadine	87	R01	C08	0.2	gsom
Olopatadine	87	R01	G03	0.2	gsom
Olopatadine	87	S01	A07	0.2	gsom
Olopatadine	87	S01	C08	0.2	gsom
Olopatadine	87	S01	G03	0.2	gsom
Olopatadine	12	S01	G03	0.2	mcode
Orphenadrine	21		R06	0.2	CL1
Oxandrolone	86	A14	C04	0.2	gsom
Oxandrolone	86	A14	N06	0.2	gsom
Oxandrolone	86	A14	N07	0.2	gsom
Oxandrolone	86	A14	S01	0.2	gsom
Oxybutynin	12	G04	G03	0.2	mcode
Oxybutynin	12	G04	S01	0.2	mcode
Paclitaxel	40	L01	A02	0.2	gsom

Phenoxybenzaı	86	C04	A14	0.2	gsom
Phenoxybenzaı	86	C04	N06	0.2	gsom
Phenoxybenzaı	86	C04	N07	0.2	gsom
Phenoxybenzaı	86	C04	S01	0.2	gsom
Phenytoin	12	N03	G03	0.2	mcode
Phenytoin	12	N03	S01	0.2	mcode
Procainamide	20	C01	B01	0.2	mcode
Procainamide	20	C01	N03	0.2	mcode
Procainamide	20	C01	N05	0.2	mcode
Procainamide	20	C01	N06	0.2	mcode
Reserpine	59	C02	L01	0.2	CL1
Reserpine	59	C02	R03	0.2	CL1
Sildenafil	60	G04	A03	0.2	CL1
Sildenafil	60	G04	C02	0.2	CL1
Sildenafil	60	G04	C03	0.2	CL1
Sildenafil	60	G04	N05	0.2	CL1
Terazosin	43	G04	C01	0.2	CL1
Terazosin	43	G04	C07	0.2	CL1
Terazosin	43	G04	N03	0.2	CL1
Terazosin	43	G04	R03	0.2	CL1
Brimonidine	41	D11	N02	0.181818	CL1
Brimonidine	41	S01	N02	0.181818	CL1
Chloramphenic	5	D06	L01	0.181818	mcode
Chloramphenic	5	D10	L01	0.181818	mcode
Chloramphenic	5	G01	L01	0.181818	mcode
Chloramphenic	5	J01	L01	0.181818	mcode
Chloramphenic	5	S01	L01	0.181818	mcode
Chloramphenic	5	S03	L01	0.181818	mcode
Dexmedetomic	41	N05	N02	0.181818	CL1
Epinephrine	41	A01	N02	0.181818	CL1
Epinephrine	41	B02	N02	0.181818	CL1
Epinephrine	41	C01	N02	0.181818	CL1
Ethosuximide	5	N03	L01	0.181818	mcode
Clotrimazole	55	A01	J02	0.176471	gsom
Clotrimazole	55	D01	J02	0.176471	gsom
Dofetilide	55	C01	D01	0.176471	gsom
Dofetilide	55	C01	G01	0.176471	gsom
Dofetilide	55	C01	J02	0.176471	gsom
Finasteride	55	G04	D01	0.176471	gsom
Finasteride	55	G04	G01	0.176471	gsom
Finasteride	55	G04	J02	0.176471	gsom
Brinzolamide	9	S01	L02	0.173913	CL1
Clotrimazole	9	A01	L02	0.173913	CL1
Clotrimazole	9	G01	L02	0.173913	CL1
Hydroxocobala	9	B03	L02	0.173913	CL1
Hydroxocobala	9	V03	L02	0.173913	CL1
Itraconazole	9	J02	L02	0.173913	CL1
Bacitracin	41	D06	C01	0.166667	gsom
Bacitracin	41	D06	C03	0.166667	gsom
Bacitracin	41	D06	C05	0.166667	gsom

Bacitracin	41	J01	C01	0.166667	gsom
Bacitracin	41	J01	C03	0.166667	gsom
Bacitracin	41	J01	C05	0.166667	gsom
Bacitracin	41	R02	C01	0.166667	gsom
Bacitracin	41	R02	C03	0.166667	gsom
Bacitracin	41	R02	C05	0.166667	gsom
Eplerenone	41	C03	C01	0.166667	gsom
Eplerenone	41	C03	C05	0.166667	gsom
Eplerenone	41	C03	D06	0.166667	gsom
Eplerenone	41	C03	J01	0.166667	gsom
Eplerenone	41	C03	R02	0.166667	gsom
Isosorbide Dini	41	C01	C03	0.166667	gsom
Isosorbide Dini	41	C01	D06	0.166667	gsom
Isosorbide Dini	41	C01	J01	0.166667	gsom
Isosorbide Dini	41	C01	R02	0.166667	gsom
Isosorbide Dini	41	C05	C03	0.166667	gsom
Isosorbide Dini	41	C05	D06	0.166667	gsom
Isosorbide Dini	41	C05	J01	0.166667	gsom
Isosorbide Dini	41	C05	R02	0.166667	gsom
Aminoglutethir	30	L02	C01	0.166667	CL1
Aminoglutethir	30	L02	L04	0.166667	CL1
Aminoglutethir	30	L02	N03	0.166667	CL1
Aminoglutethir	30	L02	N04	0.166667	CL1
Aminoglutethir	30	L02	N05	0.166667	CL1
Aripiprazole	53	N05	A03	0.166667	CL1
Aripiprazole	53	N05	A07	0.166667	CL1
Aripiprazole	53	N05	C03	0.166667	CL1
Aripiprazole	53	N05	G04	0.166667	CL1
Aripiprazole	53	N05	M03	0.166667	CL1
Atomoxetine	26	N06	A08	0.166667	mcode
Atomoxetine	26	N06	C02	0.166667	mcode
Atomoxetine	26	N06	J04	0.166667	mcode
Atomoxetine	26	N06	M04	0.166667	mcode
Atomoxetine	26	N06	N02	0.166667	mcode
Bosentan	26	C02	A08	0.166667	mcode
Bosentan	26	C02	J04	0.166667	mcode
Bosentan	26	C02	M04	0.166667	mcode
Bosentan	26	C02	N02	0.166667	mcode
Bosentan	26	C02	N06	0.166667	mcode
Dantrolene	53	M03	A03	0.166667	CL1
Dantrolene	53	M03	A07	0.166667	CL1
Dantrolene	53	M03	C03	0.166667	CL1
Dantrolene	53	M03	G04	0.166667	CL1
Dantrolene	53	M03	N05	0.166667	CL1
Doxorubicin	27	L01	G02	0.166667	CL1
Doxorubicin	27	L01	M01	0.166667	CL1
Doxorubicin	27	L01	M02	0.166667	CL1
Doxorubicin	27	L01	N03	0.166667	CL1
Doxorubicin	27	L01	N06	0.166667	CL1
Droperidol	30	N05	C01	0.166667	CL1

Droperidol	30	N05	L02	0.166667	CL1
Droperidol	30	N05	L04	0.166667	CL1
Droperidol	30	N05	N03	0.166667	CL1
Droperidol	30	N05	N04	0.166667	CL1
Entacapone	15	N04	A10	0.166667	mcode
Entacapone	15	N04	H03	0.166667	mcode
Entacapone	15	N04	L01	0.166667	mcode
Entacapone	15	N04	N05	0.166667	mcode
Entacapone	15	N04	R03	0.166667	mcode
Epinephrine	19	A01	N05	0.166667	CL1
Epinephrine	19	B02	N05	0.166667	CL1
Epinephrine	19	R01	N05	0.166667	CL1
Epinephrine	19	S01	N05	0.166667	CL1
Furosemide	53	C03	A03	0.166667	CL1
Furosemide	53	C03	A07	0.166667	CL1
Furosemide	53	C03	G04	0.166667	CL1
Furosemide	53	C03	M03	0.166667	CL1
Furosemide	53	C03	N05	0.166667	CL1
Lamotrigine	27	N03	G02	0.166667	CL1
Lamotrigine	27	N03	L01	0.166667	CL1
Lamotrigine	27	N03	M01	0.166667	CL1
Lamotrigine	27	N03	M02	0.166667	CL1
Lamotrigine	27	N03	N06	0.166667	CL1
Leflunomide	30	L04	C01	0.166667	CL1
Leflunomide	30	L04	L02	0.166667	CL1
Leflunomide	30	L04	N03	0.166667	CL1
Leflunomide	30	L04	N04	0.166667	CL1
Leflunomide	30	L04	N05	0.166667	CL1
Lithium	15	N05	A10	0.166667	mcode
Lithium	15	N05	H03	0.166667	mcode
Lithium	15	N05	L01	0.166667	mcode
Lithium	15	N05	N04	0.166667	mcode
Lithium	15	N05	R03	0.166667	mcode
Methimazole	15		A10	0.166667	mcode
Methimazole	15		H03	0.166667	mcode
Methimazole	15		L01	0.166667	mcode
Methimazole	15		N04	0.166667	mcode
Methimazole	15		N05	0.166667	mcode
Methimazole	15		R03	0.166667	mcode
Metoclopramic	53	A03	A07	0.166667	CL1
Metoclopramic	53	A03	C03	0.166667	CL1
Metoclopramic	53	A03	G04	0.166667	CL1
Metoclopramic	53	A03	M03	0.166667	CL1
Metoclopramic	53	A03	N05	0.166667	CL1
Miglitol	15	A10	H03	0.166667	mcode
Miglitol	15	A10	L01	0.166667	mcode
Miglitol	15	A10	N04	0.166667	mcode
Miglitol	15	A10	N05	0.166667	mcode
Miglitol	15	A10	R03	0.166667	mcode
Mirtazapine	27	N06	G02	0.166667	CL1

Mirtazapine	27	N06	L01	0.166667	CL1
Mirtazapine	27	N06	M01	0.166667	CL1
Mirtazapine	27	N06	M02	0.166667	CL1
Mirtazapine	27	N06	N03	0.166667	CL1
Mitotane	15	L01	A10	0.166667	mcode
Mitotane	15	L01	H03	0.166667	mcode
Mitotane	15	L01	N04	0.166667	mcode
Mitotane	15	L01	N05	0.166667	mcode
Mitotane	15	L01	R03	0.166667	mcode
Molindone	19	N05	C01	0.166667	CL1
Molindone	19	N05	R03	0.166667	CL1
Nalbuphine	26	N02	A08	0.166667	mcode
Nalbuphine	26	N02	C02	0.166667	mcode
Nalbuphine	26	N02	J04	0.166667	mcode
Nalbuphine	26	N02	M04	0.166667	mcode
Nalbuphine	26	N02	N06	0.166667	mcode
Naloxone	19	V03	C01	0.166667	CL1
Naloxone	19	V03	N05	0.166667	CL1
Naloxone	19	V03	R03	0.166667	CL1
Naproxen	27	G02	L01	0.166667	CL1
Naproxen	27	G02	N03	0.166667	CL1
Naproxen	27	G02	N06	0.166667	CL1
Naproxen	27	M01	L01	0.166667	CL1
Naproxen	27	M01	N03	0.166667	CL1
Naproxen	27	M01	N06	0.166667	CL1
Naproxen	27	M02	L01	0.166667	CL1
Naproxen	27	M02	N03	0.166667	CL1
Naproxen	27	M02	N06	0.166667	CL1
Ofloxacin	46	J01	A10	0.166667	mcode
Ofloxacin	46	J01	L01	0.166667	mcode
Ofloxacin	46	J01	N07	0.166667	mcode
Ofloxacin	46	S01	A10	0.166667	mcode
Ofloxacin	46	S01	L01	0.166667	mcode
Ofloxacin	46	S01	N07	0.166667	mcode
Ofloxacin	46	S02	A10	0.166667	mcode
Ofloxacin	46	S02	L01	0.166667	mcode
Ofloxacin	46	S02	N07	0.166667	mcode
Orlistat	26	A08	C02	0.166667	mcode
Orlistat	26	A08	J04	0.166667	mcode
Orlistat	26	A08	M04	0.166667	mcode
Orlistat	26	A08	N02	0.166667	mcode
Orlistat	26	A08	N06	0.166667	mcode
Orphenadrine	19		C01	0.166667	CL1
Orphenadrine	19		N05	0.166667	CL1
Orphenadrine	19		R03	0.166667	CL1
Oxcarbazepine	30	N03	C01	0.166667	CL1
Oxcarbazepine	30	N03	L02	0.166667	CL1
Oxcarbazepine	30	N03	L04	0.166667	CL1
Oxcarbazepine	30	N03	N04	0.166667	CL1
Oxcarbazepine	30	N03	N05	0.166667	CL1

Procainamide	30	C01	L02	0.166667	CL1
Procainamide	30	C01	L04	0.166667	CL1
Procainamide	30	C01	N03	0.166667	CL1
Procainamide	30	C01	N04	0.166667	CL1
Procainamide	30	C01	N05	0.166667	CL1
Repaglinide	46	A10	J01	0.166667	mcode
Repaglinide	46	A10	L01	0.166667	mcode
Repaglinide	46	A10	N07	0.166667	mcode
Repaglinide	46	A10	S01	0.166667	mcode
Repaglinide	46	A10	S02	0.166667	mcode
Rifabutin	26	J04	A08	0.166667	mcode
Rifabutin	26	J04	C02	0.166667	mcode
Rifabutin	26	J04	M04	0.166667	mcode
Rifabutin	26	J04	N02	0.166667	mcode
Rifabutin	26	J04	N06	0.166667	mcode
Riluzole	46	N07	A10	0.166667	mcode
Riluzole	46	N07	J01	0.166667	mcode
Riluzole	46	N07	L01	0.166667	mcode
Riluzole	46	N07	S01	0.166667	mcode
Riluzole	46	N07	S02	0.166667	mcode
Ropinirole	30	N04	C01	0.166667	CL1
Ropinirole	30	N04	L02	0.166667	CL1
Ropinirole	30	N04	L04	0.166667	CL1
Ropinirole	30	N04	N03	0.166667	CL1
Ropinirole	30	N04	N05	0.166667	CL1
Sildenafil	53	G04	A03	0.166667	CL1
Sildenafil	53	G04	A07	0.166667	CL1
Sildenafil	53	G04	C03	0.166667	CL1
Sildenafil	53	G04	M03	0.166667	CL1
Sildenafil	53	G04	N05	0.166667	CL1
Sulfasalazine	53	A07	A03	0.166667	CL1
Sulfasalazine	53	A07	C03	0.166667	CL1
Sulfasalazine	53	A07	G04	0.166667	CL1
Sulfasalazine	53	A07	M03	0.166667	CL1
Sulfasalazine	53	A07	N05	0.166667	CL1
Sulfinpyrazone	26	M04	A08	0.166667	mcode
Sulfinpyrazone	26	M04	C02	0.166667	mcode
Sulfinpyrazone	26	M04	J04	0.166667	mcode
Sulfinpyrazone	26	M04	N02	0.166667	mcode
Sulfinpyrazone	26	M04	N06	0.166667	mcode
Teniposide	46	L01	A10	0.166667	mcode
Teniposide	46	L01	J01	0.166667	mcode
Teniposide	46	L01	N07	0.166667	mcode
Teniposide	46	L01	S01	0.166667	mcode
Teniposide	46	L01	S02	0.166667	mcode
Chlorpropamid	5	A10	S01	0.153846	gsom
Ethosuximide	5	N03	S01	0.153846	gsom
Propylthiourac	5	H03	S01	0.153846	gsom
Pyrazinamide	5	J04	S01	0.153846	gsom
Amiodarone	23	C01	C02	0.153846	CL1

Amiodarone	23	C01	N01	0.153846	CL1
Amiodarone	23	C01	N07	0.153846	CL1
Atorvastatin	58	C10	G04	0.153846	CL1
Atorvastatin	58	C10	M01	0.153846	CL1
Atorvastatin	58	C10	N05	0.153846	CL1
Doxazosin	23	C02	C01	0.153846	CL1
Doxazosin	23	C02	N01	0.153846	CL1
Doxazosin	23	C02	N07	0.153846	CL1
Doxazosin	58	C02	C10	0.153846	CL1
Doxazosin	58	C02	G04	0.153846	CL1
Doxazosin	58	C02	M01	0.153846	CL1
Doxazosin	58	C02	N05	0.153846	CL1
Ketorolac	58	M01	C10	0.153846	CL1
Ketorolac	58	M01	G04	0.153846	CL1
Ketorolac	58	M01	N05	0.153846	CL1
Methadone	23	N07	C01	0.153846	CL1
Methadone	23	N07	C02	0.153846	CL1
Methadone	23	N07	N01	0.153846	CL1
Mexiletine	23	C01	C02	0.153846	CL1
Mexiletine	23	C01	N01	0.153846	CL1
Mexiletine	23	C01	N07	0.153846	CL1
Molindone	58	N05	C10	0.153846	CL1
Molindone	58	N05	G04	0.153846	CL1
Molindone	58	N05	M01	0.153846	CL1
Moricizine	23		C01	0.153846	CL1
Moricizine	23		C02	0.153846	CL1
Moricizine	23		N01	0.153846	CL1
Moricizine	23		N07	0.153846	CL1
Morphine	23	N02	C01	0.153846	CL1
Morphine	23	N02	C02	0.153846	CL1
Morphine	23	N02	N01	0.153846	CL1
Morphine	23	N02	N07	0.153846	CL1
Risperidone	58	N05	C10	0.153846	CL1
Risperidone	58	N05	G04	0.153846	CL1
Risperidone	58	N05	M01	0.153846	CL1
Adenosine	5	C01	L01	0.152941	CL1
Allopurinol	5	M04	L01	0.152941	CL1
Amifostine	5	V03	L01	0.152941	CL1
Apraclonidine	5	S01	L01	0.152941	CL1
Atovaquone	5	P01	L01	0.152941	CL1
Carbachol	5	N07	L01	0.152941	CL1
Carbachol	5	S01	L01	0.152941	CL1
Cysteamine	5		L01	0.152941	CL1
Diazoxide	5	V03	L01	0.152941	CL1
Didanosine	5	J05	L01	0.152941	CL1
Dobutamine	5	C01	L01	0.152941	CL1
Emedastine	5	S01	L01	0.152941	CL1
Tetracycline	331	A01	L01	0.142857	mcl
Tetracycline	331	D06	L01	0.142857	mcl
Tetracycline	331	J01	L01	0.142857	mcl

Tetracycline	331	S01	L01	0.142857	mcl
Tetracycline	331	S02	L01	0.142857	mcl
Tetracycline	331	S03	L01	0.142857	mcl
Vinblastine	331	L01	A01	0.142857	mcl
Vinblastine	331	L01	D06	0.142857	mcl
Vinblastine	331	L01	J01	0.142857	mcl
Vinblastine	331	L01	S01	0.142857	mcl
Vinblastine	331	L01	S02	0.142857	mcl
Vinblastine	331	L01	S03	0.142857	mcl
Adenosine	60	C01	A08	0.125	gsom
Adenosine	60	C01	C02	0.125	gsom
Adenosine	60	C01	C04	0.125	gsom
Adenosine	60	C01	D11	0.125	gsom
Adenosine	60	C01	J05	0.125	gsom
Adenosine	60	C01	M04	0.125	gsom
Adenosine	60	C01	V03	0.125	gsom
Allopurinol	60	M04	A08	0.125	gsom
Allopurinol	60	M04	C01	0.125	gsom
Allopurinol	60	M04	C02	0.125	gsom
Allopurinol	60	M04	C04	0.125	gsom
Allopurinol	60	M04	D11	0.125	gsom
Allopurinol	60	M04	J05	0.125	gsom
Allopurinol	60	M04	V03	0.125	gsom
Azelastine	28	S01	A10	0.125	CL1
Azelastine	28	S01	C03	0.125	CL1
Azelastine	28	S01	C09	0.125	CL1
Azelastine	28	S01	N03	0.125	CL1
Bumetanide	28	C03	A10	0.125	CL1
Bumetanide	28	C03	C09	0.125	CL1
Bumetanide	28	C03	N03	0.125	CL1
Bumetanide	28	C03	R06	0.125	CL1
Chlorthalidone	28		A10	0.125	CL1
Chlorthalidone	28		C03	0.125	CL1
Chlorthalidone	28		C09	0.125	CL1
Chlorthalidone	28		N03	0.125	CL1
Chlorthalidone	28		R06	0.125	CL1
Ethotoin	28	N03	A10	0.125	CL1
Ethotoin	28	N03	C03	0.125	CL1
Ethotoin	28	N03	C09	0.125	CL1
Ethotoin	28	N03	R06	0.125	CL1
Glimepiride	28	A10	C03	0.125	CL1
Glimepiride	28	A10	C09	0.125	CL1
Glimepiride	28	A10	N03	0.125	CL1
Glimepiride	28	A10	R06	0.125	CL1
Ketamine	28	N01	A10	0.125	CL1
Ketamine	28	N01	C03	0.125	CL1
Ketamine	28	N01	C09	0.125	CL1
Ketamine	28	N01	N03	0.125	CL1
Ketamine	28	N01	R06	0.125	CL1
Minoxidil	60	C02	A08	0.125	gsom

Minoxidil	60	C02	C01	0.125	gsom
Minoxidil	60	C02	C04	0.125	gsom
Minoxidil	60	C02	J05	0.125	gsom
Minoxidil	60	C02	M04	0.125	gsom
Minoxidil	60	C02	V03	0.125	gsom
Minoxidil	60	D11	A08	0.125	gsom
Minoxidil	60	D11	C01	0.125	gsom
Minoxidil	60	D11	C04	0.125	gsom
Minoxidil	60	D11	J05	0.125	gsom
Minoxidil	60	D11	M04	0.125	gsom
Minoxidil	60	D11	V03	0.125	gsom
Orlistat	60	A08	C01	0.125	gsom
Orlistat	60	A08	C02	0.125	gsom
Orlistat	60	A08	C04	0.125	gsom
Orlistat	60	A08	D11	0.125	gsom
Orlistat	60	A08	J05	0.125	gsom
Orlistat	60	A08	M04	0.125	gsom
Orlistat	60	A08	V03	0.125	gsom
Phentolamine	60	C04	A08	0.125	gsom
Phentolamine	60	C04	C01	0.125	gsom
Phentolamine	60	C04	C02	0.125	gsom
Phentolamine	60	C04	D11	0.125	gsom
Phentolamine	60	C04	J05	0.125	gsom
Phentolamine	60	C04	M04	0.125	gsom
Phentolamine	60	V03	A08	0.125	gsom
Phentolamine	60	V03	C01	0.125	gsom
Phentolamine	60	V03	C02	0.125	gsom
Phentolamine	60	V03	D11	0.125	gsom
Phentolamine	60	V03	J05	0.125	gsom
Phentolamine	60	V03	M04	0.125	gsom
Ribavirin	60	J05	A08	0.125	gsom
Ribavirin	60	J05	C01	0.125	gsom
Ribavirin	60	J05	C02	0.125	gsom
Ribavirin	60	J05	C04	0.125	gsom
Ribavirin	60	J05	D11	0.125	gsom
Ribavirin	60	J05	M04	0.125	gsom
Ribavirin	60	J05	V03	0.125	gsom
Acarbose	16	A10	N03	0.122449	CL1
Aminoglutethir	16	L02	A10	0.122449	CL1
Aminoglutethir	16	L02	N03	0.122449	CL1
Aspirin	16		A10	0.122449	CL1
Aspirin	16		N03	0.122449	CL1
Bumetanide	16	C03	A10	0.122449	CL1
Bumetanide	16	C03	N03	0.122449	CL1
Chloramphenic	16	D06	A10	0.122449	CL1
Chloramphenic	16	D06	N03	0.122449	CL1
Chlorthalidone	16		A10	0.122449	CL1
Chlorthalidone	16		N03	0.122449	CL1
Ethacrynic Acid	16		A10	0.122449	CL1
Ethacrynic Acid	16		N03	0.122449	CL1

Ethosuximide	16	N03	A10	0.122449	CL1
Ethotoin	16	N03	A10	0.122449	CL1
Methimazole	16		A10	0.122449	CL1
Methimazole	16		N03	0.122449	CL1
Argatroban	24	B01	L01	0.11828	CL1
Bacitracin	24	R02	L01	0.11828	CL1
Cefazolin	24	J01	L01	0.11828	CL1
Chloramphenic	24	G01	L01	0.11828	CL1
Chloramphenic	24	S01	L01	0.11828	CL1
Chloramphenic	24	S03	L01	0.11828	CL1
Chlorpropamid	24	A10	L01	0.11828	CL1
Chlorzoxazone	24	M03	L01	0.11828	CL1
Cysteamine	24		L01	0.11828	CL1
Dantrolene	24	M03	L01	0.11828	CL1
Didanosine	24	J05	L01	0.11828	CL1
Digoxin	24	C01	L01	0.11828	CL1
Diltiazem	24	C08	L01	0.11828	CL1
Disulfiram	24	N07	L01	0.11828	CL1
Dolasetron	24	A04	L01	0.11828	CL1
Entacapone	24	N04	L01	0.11828	CL1
Eplerenone	24	C03	L01	0.11828	CL1
Ethionamide	24	J04	L01	0.11828	CL1
Galantamine	24	N06	L01	0.11828	CL1
Glycopyrrolate	24		L01	0.11828	CL1
Hydroxyurea	24		L01	0.11828	CL1
Indapamide	24	C03	L01	0.11828	CL1
Methimazole	24		L01	0.11828	CL1
Nitroglycerin	24		L01	0.11828	CL1
Bacitracin	8	D06	C04	0.111111	mcode
Bacitracin	8	D06	M03	0.111111	mcode
Bacitracin	8	D06	N06	0.111111	mcode
Bacitracin	8	D06	N07	0.111111	mcode
Bacitracin	8	D06	P03	0.111111	mcode
Bacitracin	8	D06	S01	0.111111	mcode
Bacitracin	8	J01	C04	0.111111	mcode
Bacitracin	8	J01	M03	0.111111	mcode
Bacitracin	8	J01	N06	0.111111	mcode
Bacitracin	8	J01	N07	0.111111	mcode
Bacitracin	8	J01	P03	0.111111	mcode
Bacitracin	8	J01	S01	0.111111	mcode
Bacitracin	8	R02	C04	0.111111	mcode
Bacitracin	8	R02	M03	0.111111	mcode
Bacitracin	8	R02	N06	0.111111	mcode
Bacitracin	8	R02	N07	0.111111	mcode
Bacitracin	8	R02	P03	0.111111	mcode
Bacitracin	8	R02	S01	0.111111	mcode
Caffeine	8	N06	C04	0.111111	mcode
Caffeine	8	N06	D06	0.111111	mcode
Caffeine	8	N06	J01	0.111111	mcode
Caffeine	8	N06	M03	0.111111	mcode

Caffeine	8	N06	N07	0.111111	mcode
Caffeine	8	N06	P03	0.111111	mcode
Caffeine	8	N06	R02	0.111111	mcode
Caffeine	8	N06	S01	0.111111	mcode
Carbachol	8	N07	C04	0.111111	mcode
Carbachol	8	N07	D06	0.111111	mcode
Carbachol	8	N07	J01	0.111111	mcode
Carbachol	8	N07	M03	0.111111	mcode
Carbachol	8	N07	N06	0.111111	mcode
Carbachol	8	N07	P03	0.111111	mcode
Carbachol	8	N07	R02	0.111111	mcode
Carbachol	8	S01	C04	0.111111	mcode
Carbachol	8	S01	D06	0.111111	mcode
Carbachol	8	S01	J01	0.111111	mcode
Carbachol	8	S01	M03	0.111111	mcode
Carbachol	8	S01	N06	0.111111	mcode
Carbachol	8	S01	P03	0.111111	mcode
Carbachol	8	S01	R02	0.111111	mcode
Chlorzoxazone	8	M03	C04	0.111111	mcode
Chlorzoxazone	8	M03	D06	0.111111	mcode
Chlorzoxazone	8	M03	J01	0.111111	mcode
Chlorzoxazone	8	M03	N06	0.111111	mcode
Chlorzoxazone	8	M03	N07	0.111111	mcode
Chlorzoxazone	8	M03	P03	0.111111	mcode
Chlorzoxazone	8	M03	R02	0.111111	mcode
Chlorzoxazone	8	M03	S01	0.111111	mcode
Malathion	8	P03	C04	0.111111	mcode
Malathion	8	P03	D06	0.111111	mcode
Malathion	8	P03	J01	0.111111	mcode
Malathion	8	P03	M03	0.111111	mcode
Malathion	8	P03	N06	0.111111	mcode
Malathion	8	P03	N07	0.111111	mcode
Malathion	8	P03	R02	0.111111	mcode
Malathion	8	P03	S01	0.111111	mcode
Phenoxybenzar	8	C04	D06	0.111111	mcode
Phenoxybenzar	8	C04	J01	0.111111	mcode
Phenoxybenzar	8	C04	M03	0.111111	mcode
Phenoxybenzar	8	C04	N06	0.111111	mcode
Phenoxybenzar	8	C04	N07	0.111111	mcode
Phenoxybenzar	8	C04	P03	0.111111	mcode
Phenoxybenzar	8	C04	R02	0.111111	mcode
Phenoxybenzar	8	C04	S01	0.111111	mcode
Allopurinol	417	M04	L01	0.103627	mcl
Amifostine	417	V03	L01	0.103627	mcl
Amiodarone	417	C01	L01	0.103627	mcl
Apraclonidine	417	S01	L01	0.103627	mcl
Bacitracin	417	J01	L01	0.103627	mcl
Baclofen	417	M03	L01	0.103627	mcl
Bosentan	417	C02	L01	0.103627	mcl
Caffeine	417	N06	L01	0.103627	mcl

Chloramphenic	417	G01	L01	0.103627	mcl
Chloramphenic	417	J01	L01	0.103627	mcl
Ciprofloxacin	417	S02	L01	0.103627	mcl
Clomipramine	417	N06	L01	0.103627	mcl
Cysteamine	417		L01	0.103627	mcl
Diazoxide	417	C02	L01	0.103627	mcl
Glycopyrrolate	417		L01	0.103627	mcl
Hydroxyurea	417		L01	0.103627	mcl
Methimazole	417		L01	0.103627	mcl
Moricizine	417		L01	0.103627	mcl
Nitroglycerin	417		L01	0.103627	mcl
Acetazolamide	0	S01	N05	0.071856	mcode
Acitretin	0	D05	N05	0.071856	mcode
Bicalutamide	0	L02	N05	0.071856	mcode
Bromocriptine	0	N04	N05	0.071856	mcode
Capecitabine	0	L01	N05	0.071856	mcode
Celecoxib	0	M01	N05	0.071856	mcode
Chlorthalidone	0		N05	0.071856	mcode
Clotrimazole	0	A01	N05	0.071856	mcode
Desloratadine	0	R06	N05	0.071856	mcode
Ethacrynic Acid	0		N05	0.071856	mcode
Cysteamine	13		A01	0.055556	mcode
Cysteamine	13		A14	0.055556	mcode
Cysteamine	13		B01	0.055556	mcode
Cysteamine	13		B02	0.055556	mcode
Cysteamine	13		C02	0.055556	mcode
Cysteamine	13		D06	0.055556	mcode
Cysteamine	13		J01	0.055556	mcode
Cysteamine	13		J05	0.055556	mcode
Cysteamine	13		L01	0.055556	mcode
Cysteamine	13		N03	0.055556	mcode
Cysteamine	13		N04	0.055556	mcode
Cysteamine	13		N07	0.055556	mcode
Cysteamine	13		P03	0.055556	mcode
Cysteamine	13		R07	0.055556	mcode
Cysteamine	13		S01	0.055556	mcode
Cysteamine	13		S02	0.055556	mcode
Cysteamine	13		S03	0.055556	mcode
Cysteamine	13		V03	0.055556	mcode
Diazoxide	13	C02	A01	0.055556	mcode
Diazoxide	13	C02	A14	0.055556	mcode
Diazoxide	13	C02	B01	0.055556	mcode
Diazoxide	13	C02	B02	0.055556	mcode
Diazoxide	13	C02	D06	0.055556	mcode
Diazoxide	13	C02	J01	0.055556	mcode
Diazoxide	13	C02	J05	0.055556	mcode
Diazoxide	13	C02	L01	0.055556	mcode
Diazoxide	13	C02	N03	0.055556	mcode
Diazoxide	13	C02	N04	0.055556	mcode
Diazoxide	13	C02	N07	0.055556	mcode

Diazoxide	13	C02	P03	0.055556	mcode
Diazoxide	13	C02	R07	0.055556	mcode
Diazoxide	13	C02	S01	0.055556	mcode
Diazoxide	13	C02	S02	0.055556	mcode
Diazoxide	13	C02	S03	0.055556	mcode
Diazoxide	13	V03	A01	0.055556	mcode
Diazoxide	13	V03	A14	0.055556	mcode
Diazoxide	13	V03	B01	0.055556	mcode
Diazoxide	13	V03	B02	0.055556	mcode
Diazoxide	13	V03	D06	0.055556	mcode
Diazoxide	13	V03	J01	0.055556	mcode
Diazoxide	13	V03	J05	0.055556	mcode
Diazoxide	13	V03	L01	0.055556	mcode
Diazoxide	13	V03	N03	0.055556	mcode
Diazoxide	13	V03	N04	0.055556	mcode
Diazoxide	13	V03	N07	0.055556	mcode
Diazoxide	13	V03	P03	0.055556	mcode
Diazoxide	13	V03	R07	0.055556	mcode
Diazoxide	13	V03	S01	0.055556	mcode
Diazoxide	13	V03	S02	0.055556	mcode
Diazoxide	13	V03	S03	0.055556	mcode
Didanosine	13	J05	A01	0.055556	mcode
Didanosine	13	J05	A14	0.055556	mcode
Didanosine	13	J05	B01	0.055556	mcode
Didanosine	13	J05	B02	0.055556	mcode
Didanosine	13	J05	C02	0.055556	mcode
Didanosine	13	J05	D06	0.055556	mcode
Didanosine	13	J05	J01	0.055556	mcode
Didanosine	13	J05	L01	0.055556	mcode
Didanosine	13	J05	N03	0.055556	mcode
Didanosine	13	J05	N04	0.055556	mcode
Didanosine	13	J05	N07	0.055556	mcode
Didanosine	13	J05	P03	0.055556	mcode
Didanosine	13	J05	R07	0.055556	mcode
Didanosine	13	J05	S01	0.055556	mcode
Didanosine	13	J05	S02	0.055556	mcode
Didanosine	13	J05	S03	0.055556	mcode
Didanosine	13	J05	V03	0.055556	mcode
Disulfiram	13	N07	A01	0.055556	mcode
Disulfiram	13	N07	A14	0.055556	mcode
Disulfiram	13	N07	B01	0.055556	mcode
Disulfiram	13	N07	B02	0.055556	mcode
Disulfiram	13	N07	C02	0.055556	mcode
Disulfiram	13	N07	D06	0.055556	mcode
Disulfiram	13	N07	J01	0.055556	mcode
Disulfiram	13	N07	J05	0.055556	mcode
Disulfiram	13	N07	L01	0.055556	mcode
Disulfiram	13	N07	N03	0.055556	mcode
Disulfiram	13	N07	N04	0.055556	mcode
Disulfiram	13	N07	R07	0.055556	mcode

Disulfiram	13	N07	S01	0.055556	mcode
Disulfiram	13	N07	S02	0.055556	mcode
Disulfiram	13	N07	S03	0.055556	mcode
Disulfiram	13	N07	V03	0.055556	mcode
Disulfiram	13	P03	A01	0.055556	mcode
Disulfiram	13	P03	A14	0.055556	mcode
Disulfiram	13	P03	B01	0.055556	mcode
Disulfiram	13	P03	B02	0.055556	mcode
Disulfiram	13	P03	C02	0.055556	mcode
Disulfiram	13	P03	D06	0.055556	mcode
Disulfiram	13	P03	J01	0.055556	mcode
Disulfiram	13	P03	J05	0.055556	mcode
Disulfiram	13	P03	L01	0.055556	mcode
Disulfiram	13	P03	N03	0.055556	mcode
Disulfiram	13	P03	N04	0.055556	mcode
Disulfiram	13	P03	R07	0.055556	mcode
Disulfiram	13	P03	S01	0.055556	mcode
Disulfiram	13	P03	S02	0.055556	mcode
Disulfiram	13	P03	S03	0.055556	mcode
Disulfiram	13	P03	V03	0.055556	mcode
Gefitinib	13	L01	A01	0.055556	mcode
Gefitinib	13	L01	A14	0.055556	mcode
Gefitinib	13	L01	B01	0.055556	mcode
Gefitinib	13	L01	B02	0.055556	mcode
Gefitinib	13	L01	C02	0.055556	mcode
Gefitinib	13	L01	D06	0.055556	mcode
Gefitinib	13	L01	J01	0.055556	mcode
Gefitinib	13	L01	J05	0.055556	mcode
Gefitinib	13	L01	N03	0.055556	mcode
Gefitinib	13	L01	N04	0.055556	mcode
Gefitinib	13	L01	N07	0.055556	mcode
Gefitinib	13	L01	P03	0.055556	mcode
Gefitinib	13	L01	R07	0.055556	mcode
Gefitinib	13	L01	S01	0.055556	mcode
Gefitinib	13	L01	S02	0.055556	mcode
Gefitinib	13	L01	S03	0.055556	mcode
Gefitinib	13	L01	V03	0.055556	mcode
Mephenytoin	13	N03	A01	0.055556	mcode
Mephenytoin	13	N03	A14	0.055556	mcode
Mephenytoin	13	N03	B01	0.055556	mcode
Mephenytoin	13	N03	B02	0.055556	mcode
Mephenytoin	13	N03	C02	0.055556	mcode
Mephenytoin	13	N03	D06	0.055556	mcode
Mephenytoin	13	N03	J01	0.055556	mcode
Mephenytoin	13	N03	J05	0.055556	mcode
Mephenytoin	13	N03	L01	0.055556	mcode
Mephenytoin	13	N03	N04	0.055556	mcode
Mephenytoin	13	N03	N07	0.055556	mcode
Mephenytoin	13	N03	P03	0.055556	mcode
Mephenytoin	13	N03	R07	0.055556	mcode

Mephenytoin	13	N03	S01	0.055556	mcode
Mephenytoin	13	N03	S02	0.055556	mcode
Mephenytoin	13	N03	S03	0.055556	mcode
Mephenytoin	13	N03	V03	0.055556	mcode
Nitric Oxide	13	R07	A01	0.055556	mcode
Nitric Oxide	13	R07	A14	0.055556	mcode
Nitric Oxide	13	R07	B01	0.055556	mcode
Nitric Oxide	13	R07	B02	0.055556	mcode
Nitric Oxide	13	R07	C02	0.055556	mcode
Nitric Oxide	13	R07	D06	0.055556	mcode
Nitric Oxide	13	R07	J01	0.055556	mcode
Nitric Oxide	13	R07	J05	0.055556	mcode
Nitric Oxide	13	R07	L01	0.055556	mcode
Nitric Oxide	13	R07	N03	0.055556	mcode
Nitric Oxide	13	R07	N04	0.055556	mcode
Nitric Oxide	13	R07	N07	0.055556	mcode
Nitric Oxide	13	R07	P03	0.055556	mcode
Nitric Oxide	13	R07	S01	0.055556	mcode
Nitric Oxide	13	R07	S02	0.055556	mcode
Nitric Oxide	13	R07	S03	0.055556	mcode
Nitric Oxide	13	R07	V03	0.055556	mcode
Oxandrolone	13	A14	A01	0.055556	mcode
Oxandrolone	13	A14	B01	0.055556	mcode
Oxandrolone	13	A14	B02	0.055556	mcode
Oxandrolone	13	A14	C02	0.055556	mcode
Oxandrolone	13	A14	D06	0.055556	mcode
Oxandrolone	13	A14	J01	0.055556	mcode
Oxandrolone	13	A14	J05	0.055556	mcode
Oxandrolone	13	A14	L01	0.055556	mcode
Oxandrolone	13	A14	N03	0.055556	mcode
Oxandrolone	13	A14	N04	0.055556	mcode
Oxandrolone	13	A14	N07	0.055556	mcode
Oxandrolone	13	A14	P03	0.055556	mcode
Oxandrolone	13	A14	R07	0.055556	mcode
Oxandrolone	13	A14	S01	0.055556	mcode
Oxandrolone	13	A14	S02	0.055556	mcode
Oxandrolone	13	A14	S03	0.055556	mcode
Oxandrolone	13	A14	V03	0.055556	mcode
Procyclidine	13	N04	A01	0.055556	mcode
Procyclidine	13	N04	A14	0.055556	mcode
Procyclidine	13	N04	B01	0.055556	mcode
Procyclidine	13	N04	B02	0.055556	mcode
Procyclidine	13	N04	C02	0.055556	mcode
Procyclidine	13	N04	D06	0.055556	mcode
Procyclidine	13	N04	J01	0.055556	mcode
Procyclidine	13	N04	J05	0.055556	mcode
Procyclidine	13	N04	L01	0.055556	mcode
Procyclidine	13	N04	N03	0.055556	mcode
Procyclidine	13	N04	N07	0.055556	mcode
Procyclidine	13	N04	P03	0.055556	mcode

Procyclidine	13	N04	R07	0.055556	mcode
Procyclidine	13	N04	S01	0.055556	mcode
Procyclidine	13	N04	S02	0.055556	mcode
Procyclidine	13	N04	S03	0.055556	mcode
Procyclidine	13	N04	V03	0.055556	mcode
Tetracycline	13	A01	A14	0.055556	mcode
Tetracycline	13	A01	B01	0.055556	mcode
Tetracycline	13	A01	B02	0.055556	mcode
Tetracycline	13	A01	C02	0.055556	mcode
Tetracycline	13	A01	J05	0.055556	mcode
Tetracycline	13	A01	L01	0.055556	mcode
Tetracycline	13	A01	N03	0.055556	mcode
Tetracycline	13	A01	N04	0.055556	mcode
Tetracycline	13	A01	N07	0.055556	mcode
Tetracycline	13	A01	P03	0.055556	mcode
Tetracycline	13	A01	R07	0.055556	mcode
Tetracycline	13	A01	V03	0.055556	mcode
Tetracycline	13	D06	A14	0.055556	mcode
Tetracycline	13	D06	B01	0.055556	mcode
Tetracycline	13	D06	B02	0.055556	mcode
Tetracycline	13	D06	C02	0.055556	mcode
Tetracycline	13	D06	J05	0.055556	mcode
Tetracycline	13	D06	L01	0.055556	mcode
Tetracycline	13	D06	N03	0.055556	mcode
Tetracycline	13	D06	N04	0.055556	mcode
Tetracycline	13	D06	N07	0.055556	mcode
Tetracycline	13	D06	P03	0.055556	mcode
Tetracycline	13	D06	R07	0.055556	mcode
Tetracycline	13	D06	V03	0.055556	mcode
Tetracycline	13	J01	A14	0.055556	mcode
Tetracycline	13	J01	B01	0.055556	mcode
Tetracycline	13	J01	B02	0.055556	mcode
Tetracycline	13	J01	C02	0.055556	mcode
Tetracycline	13	J01	J05	0.055556	mcode
Tetracycline	13	J01	L01	0.055556	mcode
Tetracycline	13	J01	N03	0.055556	mcode
Tetracycline	13	J01	N04	0.055556	mcode
Tetracycline	13	J01	N07	0.055556	mcode
Tetracycline	13	J01	P03	0.055556	mcode
Tetracycline	13	J01	R07	0.055556	mcode
Tetracycline	13	J01	V03	0.055556	mcode
Tetracycline	13	S01	A14	0.055556	mcode
Tetracycline	13	S01	B01	0.055556	mcode
Tetracycline	13	S01	B02	0.055556	mcode
Tetracycline	13	S01	C02	0.055556	mcode
Tetracycline	13	S01	J05	0.055556	mcode
Tetracycline	13	S01	L01	0.055556	mcode
Tetracycline	13	S01	N03	0.055556	mcode
Tetracycline	13	S01	N04	0.055556	mcode
Tetracycline	13	S01	N07	0.055556	mcode

Tetracycline	13	S01	P03	0.055556	mcode
Tetracycline	13	S01	R07	0.055556	mcode
Tetracycline	13	S01	V03	0.055556	mcode
Tetracycline	13	S02	A14	0.055556	mcode
Tetracycline	13	S02	B01	0.055556	mcode
Tetracycline	13	S02	B02	0.055556	mcode
Tetracycline	13	S02	C02	0.055556	mcode
Tetracycline	13	S02	J05	0.055556	mcode
Tetracycline	13	S02	L01	0.055556	mcode
Tetracycline	13	S02	N03	0.055556	mcode
Tetracycline	13	S02	N04	0.055556	mcode
Tetracycline	13	S02	N07	0.055556	mcode
Tetracycline	13	S02	P03	0.055556	mcode
Tetracycline	13	S02	R07	0.055556	mcode
Tetracycline	13	S02	V03	0.055556	mcode
Tetracycline	13	S03	A14	0.055556	mcode
Tetracycline	13	S03	B01	0.055556	mcode
Tetracycline	13	S03	B02	0.055556	mcode
Tetracycline	13	S03	C02	0.055556	mcode
Tetracycline	13	S03	J05	0.055556	mcode
Tetracycline	13	S03	L01	0.055556	mcode
Tetracycline	13	S03	N03	0.055556	mcode
Tetracycline	13	S03	N04	0.055556	mcode
Tetracycline	13	S03	N07	0.055556	mcode
Tetracycline	13	S03	P03	0.055556	mcode
Tetracycline	13	S03	R07	0.055556	mcode
Tetracycline	13	S03	V03	0.055556	mcode
