# Supplementary material for: A two-tiered unsupervised clustering approach for drug repositioning through heterogeneous data integration
Source: BMC Bioinformatics. 2018 Apr 11;19:129. doi: 10.1186/s12859-018-2123-4 (PMC5896044; doi:10.1186/s12859-018-2123-4)
Supplement: Supplementary file 5 — The ATC code list. This includes the ATC codes (Second Level) and the corresponding ATC therapeutic class names. (PDF 363 kb) [file 12859_2018_2123_MOESM5_ESM.pdf]

ATC code	ATC therapeutic class name
A01	STOMATOLOGICAL PREPARATIONS
A02	DRUGS FOR ACID RELATED DISORDERS
A03	DRUGS FOR FUNCTIONAL GASTROINTESTINAL DISORDERS
A04	ANTIEMETICS AND ANTINAUSEANTS
A05	BILE AND LIVER THERAPY
A06	DRUGS FOR CONSTIPATION
A07	ANTIDIARRHEALS, INTESTINAL ANTIINFLAMMATORY/ANTIINFECTIVE AGENTS
A08	ANTIOBESITY PREPARATIONS, EXCL. DIET PRODUCTS
A09	DIGESTIVES, INCL. ENZYMES
A10	DRUGS USED IN DIABETES
A11	VITAMINS
A12	MINERAL SUPPLEMENTS
A13	TONICS
A14	ANABOLIC AGENTS FOR SYSTEMIC USE
A15	APPETITE STIMULANTS
A16	OTHER ALIMENTARY TRACT AND METABOLISM PRODUCTS
B01	ANTITHROMBOTIC AGENTS
B02	ANTIHEMORRHAGICS
B03	ANTIANEMIC PREPARATIONS
B05	BLOOD SUBSTITUTES AND PERFUSION SOLUTIONS
B06	OTHER HEMATOLOGICAL AGENTS
C01	CARDIAC THERAPY
C02	ANTIHYPERTENSIVES
C03	DIURETICS
C04	PERIPHERAL VASODILATORS
C05	VASOPROTECTIVES
C07	BETA BLOCKING AGENTS
C08	CALCIUM CHANNEL BLOCKERS
C09	AGENTS ACTING ON THE RENIN-ANGIOTENSIN SYSTEM
C10	LIPID MODIFYING AGENTS
D01	ANTIFUNGALS FOR DERMATOLOGICAL USE
D02	EMOLLIENTS AND PROTECTIVES
D03	PREPARATIONS FOR TREATMENT OF WOUNDS AND ULCERS
D04	ANTIPRURITICS, INCL. ANTIHISTAMINES, ANESTHETICS, ETC.
D05	ANTIPSORIATICS
D06	ANTIBIOTICS AND CHEMOTHERAPEUTICS FOR DERMATOLOGICAL USE
D07	CORTICOSTEROIDS, DERMATOLOGICAL PREPARATIONS
D08	ANTISEPTICS AND DISINFECTANTS
D09	MEDICATED DRESSINGS
D10	ANTI-ACNE PREPARATIONS
D11	OTHER DERMATOLOGICAL PREPARATIONS
G01	GYNECOLOGICAL ANTIINFECTIVES AND ANTISEPTICS
G02	OTHER GYNECOLOGICALS
G03	SEX HORMONES AND MODULATORS OF THE GENITAL SYSTEM
G04	UROLOGICALS
H01	PITUITARY AND HYPOTHALAMIC HORMONES AND ANALOGUES
H02	CORTICOSTEROIDS FOR SYSTEMIC USE
H03	THYROID THERAPY
H04	PANCREATIC HORMONES

H05	CALCIUM HOMEOSTASIS
J01	ANTIBACTERIALS FOR SYSTEMIC USE
J02	ANTIMYCOTICS FOR SYSTEMIC USE
J04	ANTIMYCOBACTERIALS
J05	ANTIVIRALS FOR SYSTEMIC USE
J06	IMMUNE SERA AND IMMUNOGLOBULINS
J07	VACCINES
L01	ANTINEOPLASTIC AGENTS
L02	ENDOCRINE THERAPY
L03	IMMUNOSTIMULANTS
L04	IMMUNOSUPPRESSANTS
M01	ANTIINFLAMMATORY AND ANTIRHEUMATIC PRODUCTS
M02	TOPICAL PRODUCTS FOR JOINT AND MUSCULAR PAIN
M03	MUSCLE RELAXANTS
M04	ANTIGOUT PREPARATIONS
M05	DRUGS FOR TREATMENT OF BONE DISEASES
M09	OTHER DRUGS FOR DISORDERS OF THE MUSCULO-SKELETAL SYSTEM
N01	ANESTHETICS
N02	ANALGESICS
N03	ANTIEPILEPTICS
N04	ANTI-PARKINSON DRUGS
N05	PSYCHOLEPTICS
N06	PSYCHOANALEPTICS
N07	OTHER NERVOUS SYSTEM DRUGS
P01	ANTIPROTOZOALS
P02	ANTHELMINTICS
P03	ECTOPARASITICIDES, INCL. SCABICIDES, INSECTICIDES AND REPELLENTS
R01	NASAL PREPARATIONS
R02	THROAT PREPARATIONS
R03	DRUGS FOR OBSTRUCTIVE AIRWAY DISEASES
R05	COUGH AND COLD PREPARATIONS
R06	ANTIHISTAMINES FOR SYSTEMIC USE
R07	OTHER RESPIRATORY SYSTEM PRODUCTS
S01	OPHTHALMOLOGICALS
S02	OTOLOGICALS
S03	OPHTHALMOLOGICAL AND OTOLOGICAL PREPARATIONS
V01	ALLERGENS
V03	ALL OTHER THERAPEUTIC PRODUCTS
V04	DIAGNOSTIC AGENTS
V06	GENERAL NUTRIENTS
V07	ALL OTHER NON-THERAPEUTIC PRODUCTS
V08	CONTRAST MEDIA
V09	DIAGNOSTIC RADIOPHARMACEUTICALS
V10	THERAPEUTIC RADIOPHARMACEUTICALS
V20	SURGICAL DRESSINGS
